# A pH-responsive nanoplatform with dual-modality imaging for enhanced cancer phototherapy and diagnosis of lung metastasis

**DOI:** 10.1186/s12951-024-02431-6

**Published:** 2024-04-15

**Authors:** Mujie Yuan, Zeyu Han, Yan Li, Xin Zhan, Yong Sun, Bin He, Yan Liang, Kui Luo, Fan Li

**Affiliations:** 1 Department of Oral Implantology, The Affiliated Hospital of Qingdao University, Qingdao, 266000 China; 2 Precision Research Center for Refractory Diseases, Shanghai General Hospital, Shanghai Jiao Tong University School of Medicine, Shanghai, 200080 China; 3 Department of Pharmaceutics, Qingdao University School of Pharmacy, Qingdao, 266021 China; 4 National Engineering Research Center for Biomaterials, Sichuan University, Chengdu, 610064 China; 5 Huaxi MR Research Center (HMRRC), Department of Radiology, West China Hospital, Sichuan University, Chengdu, 610041 China

**Keywords:** Enhanced phototherapy, Dual-modality fluorescence/^19^F MRI, pH-responsive, Lung metastasis

## Abstract

To address the limitations of traditional photothermal therapy (PTT)/ photodynamic therapy (PDT) and real-time cancer metastasis detection, a pH-responsive nanoplatform (NP) with dual-modality imaging capability was rationally designed. Herein, 1 H,1 H-undecafluorohexylamine (PFC), served as both an oxygen carrier and a ^19^F magnetic resonance imaging (MRI) probe, and photosensitizer indocyanine green (ICG) were grafted onto the pH-responsive peptide hexahistidine (H_6_) to form H_6_-PFC-ICG (HPI). Subsequently, the heat shock protein 90 inhibitor, gambogic acid (GA), was incorporated into hyaluronic acid (HA) modified HPI (HHPI), yielding the ultimate HHPI@GA NPs. Upon self-assembly, HHPI@GA NPs passively accumulated in tumor tissues, facilitating oxygen release and HA-mediated cell uptake. Once phagocytosed by lysosomes, protonation of H_6_ was triggered due to the low pH, resulting in the release of GA. With near-infrared laser irradiation, GA-mediated decreased HSP90 expression and PFC-mediated increased ROS generation amplified the PTT/PDT effect of HHPI@GA, leading to excellent in vitro and in vivo anticancer efficacies. Additionally, the fluorescence and ^19^F MRI dual-imaging capabilities of HHPI@GA NPs enabled effective real-time primary cancer and lung metastasis monitoring. This work offers a novel approach for enhanced cancer phototherapy, as well as precise cancer diagnosis.

**Supplementary Information:**

The online version contains supplementary material available at 10.1186/s12951-024-02431-6.

## Introduction

Compared to conventional therapeutic approaches, phototherapy garners considerable attention for cancer treatment due to its noninvasive nature, precise spatiotemporal selectivity, and minimal side effects [1, 2]. Phototherapy is mainly divided into photothermal therapy (PTT) and photodynamic therapy (PDT) [3–5], PTT refers to photoabsorbing agents absorb light energy to generate heat ablating cancer cells [6, 7], while PDT utilizes photosensitizers to transfer light energy to surrounding oxygen, triggering toxic reactive oxygen species (ROS) to kill cancer cells [8]. Although PTT or PDT has made great progress in cancer treatment recent years, it still faces formidable and ongoing challenges due to the complexity of tumor microenvironment (TME) [9]. Specifically, hyperthermia induced by traditional PTT could effectively kill cancer cells, but after a certain time, cancer cells will overexpress heat shock proteins (HSPs) and acquire thermotolerance against PTT [10]. For traditional PDT, hypoxia is the main characteristic of TME [11], resulting in insufficient ROS generation and limiting therapeutic efficacy of the highly oxygen-dependent PDT [12]. Hence, it is imperative to develop an efficient strategy to overcome above difficulties and enhance the therapeutic effect of phototherapy.

At present, the methods improving phototherapy could be roughly divided into two aspects. For PTT, a series of HSPs inhibitors, such as gambogic acid (GA), tanespimycin, and ganetespib, were used to down-regulated the expression of HSPs, thereby restoring PTT sensitivity of cells [13, 14]. The latest research has demonstrated that GA can effectively restore the sensitivity of cells to heat because of its highly inhibitory effect on the heat shock protein 90 (HSP90) [15]. For PDT, alleviating tumor hypoxia was the mainstream solution to increase ROS generation of PDT [16, 17]. Delivering exogenous oxygen to tumor directly, generating oxygen in situ, and reducing tumor cellular oxygen consumption by inhibiting cellular aerobic respiration were commonly used strategies to alleviate hypoxia [18, 19]. Perfluorocarbons (PFC) is an oxygen carrying agent known for high oxygen solubility and excellent biocompatibility [20, 21]. A variety of PFC and photosensitizer co-loaded nanodroplets or hollow inorganic nanoplatforms (NPs) has been developed to supply oxygen to tumor tissues and improve PDT effect [22].

These methods have significantly advanced the efficacy of phototherapy for treating primary cancers; however, they prove inadequate for cancers with a propensity for metastasis [23, 24]. Despite successful treatment at the primary site, metastatic cancers can progressively invade and jeopardize the patient’s life [25, 26]. Therefore, alongside improving the treatment of primary lesions, monitoring cancer metastasis is crucial [27, 28]. In recent years, various NPs with diagnostic capabilities have emerged to detect metastatic cancers using techniques such as fluorescence imaging, magnetic resonance imaging (MRI), photoacoustic imaging and et al. [29–31]. The combined application of multiple imaging modalities not only enhances diagnostic accuracy and cancer localization but also effectively tracks and monitors cancer progression in real time, contributing to the development of more personalized and effective treatment plans [32–34].

Indocyanine green (ICG) is a near-infrared (NIR) fluorescent dye approved for clinical use by the U.S. Food and Drug Administration (FDA) [35]. It exhibits effective PTT and PDT effects under NIR light irradiation, thus effectively ablating cancers [36, 37]. Importantly, ICG emits fluorescence when excited by a laser, making it a valuable imaging probe [38, 39]. Additionally, PFCs, serving as oxygen carriers, exhibit a significant ^19^F chemical shift, enabling them to act as ^19^F MRI probes [35, 40]. Given the near absence of ^19^F in the human body, ^19^F MRI not only retains the advantages of ^1^H MRI but also features extremely low background noise, offering unique benefits in cancer diagnosis, particularly in detecting metastatic cancers [41–43].

**Scheme 1:**
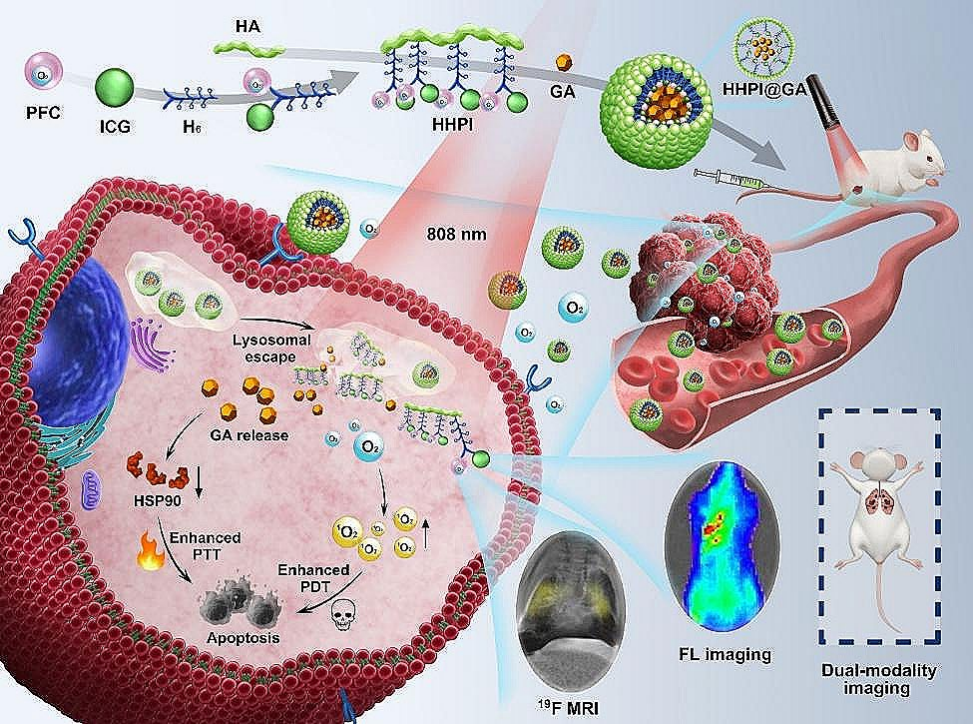
Illustration of HHPI@GA NPs for enhanced phototherapy and dual-modality imaging of cancer metastasis

In this study, we engineered NPs with good biocompatibility for enhanced phototherapy against cancer and dual-modality imaging diagnosis of metastases. As illustrated in Scheme 1, oxygen donor and ^19^F MRI probe PFC as well as the photosensitizer ICG were initially linked by the hexahistidine (H_6_) to form H_6_-PFC-ICG (HPI). Subsequently, HPI was grafted onto hyaluronic acid (HA) via Michael addition to synthesize HA-H_6_-PFC-ICG (HHPI). Finally, HHPI@GA NPs were formed by self-assembly with GA in deionized (DI) water. After preparation, HHPI@GA NPs were intravenously injected into tumor-bearing mouse; the NPs accumulated in tumor tissues via the enhanced permeability and retention (EPR) effect and HA-mediated active targeting, releasing oxygen and regulating local hypoxic conditions. Once the NPs were endocytosed by cancer cells and exposed to an acidic lysosomal environment, the protonation of H_6_ was triggered due to the low pH, leading to the release of GA and inhibition of HSP90 expression. With NIR laser irradiation, enhanced PTT and PDT were achieved to improve therapeutic effects on cancer. In addition, ICG and PFC can be respectively used as fluorescent and ^19^F MR imaging probe to indicate the primary cancer and lung metastasis. Thus, HHPI@GA NPs not only augmented the anticancer effect but also monitored cancer progression in real time, providing a promising strategy for cancer theranostics.

## Results and discussion

### Characterization of HHPI and HHPI@GA

The synthetic scheme for the amphiphile HHPI is detailed in **Fig. ****S1**. During synthesis, the successful creation of various intermediate and final products was confirmed through mass spectrum, GPC, and FTIR. Mass spectrum results validated the molecular weights of the intermediates: PFC-Mal (*m/z* [M + H]^+^: 451.05087), H_6_-PFC (*m/z* [M + 2H]^2^^+^: 872.30531; [M + 3H]^3^^+^: 581.87263; [M + 4H]^4^^+^: 436.65631), H_6_-PFC-ICG (*m/z* [M + 3H]^3^^+^: 779.32108; [M + 4H]^4+^: 584.74264; [M + 5H]^5^^+^: 467.99557), NH_2_-H_6_-PFC-ICG (*m/z* [M + 3H]^3^^+^: 705.29839; [M + 4H]^4+^: 529.22562; [M + 5H]^5^^+^: 423.58196), and Mal-H_6_-PFC-ICG (*m/z* [M + 3H]^3^^+^: 755.64071; [M + 4H]^4^^+^: 566.98236; [M + 5H]^5+^: 453.78735), confirming the accuracy of their molecular masses **(Fig. S2-6)**. The FTIR spectra of HA, HPI and HHPI was demonstrated in **Fig. ****S1****2A**. The bands at 1409 cm^– 1^ and 928 cm^– 1^were attributed to C-N and C = C stretching of HPI respectively, confirming the successful synthesis of HHPI. In addition, the GPC result **(Fig. ****S1****2B)** showed only one peak in the HHPI spectra without other impurity peaks, indicating the absence of any residual HA or HPI. The outflow times of the three compounds further verified the successful HHPI synthesis. Furthermore, based on the similar synthesis steps and characterization results, HHHI was confirmed to be successfully synthesized **(Fig. S7-11 and Fig. ****S1****2A-B).**

### Physicochemical properties of HHPI and HHPI@GA

Upon being transferred from DMF to DI water, the amphiphiles HHPI and HHPI@GA self-assembled into NPs. GA was encapsulated into NPs through hydrophobic interactions, with an encapsulation efficiency of 65.17 ± 1.34% and drug loading content of 3.1 ± 0.06% calculated by standard curve **(Fig. S12C)**.

**Fig. 1:**
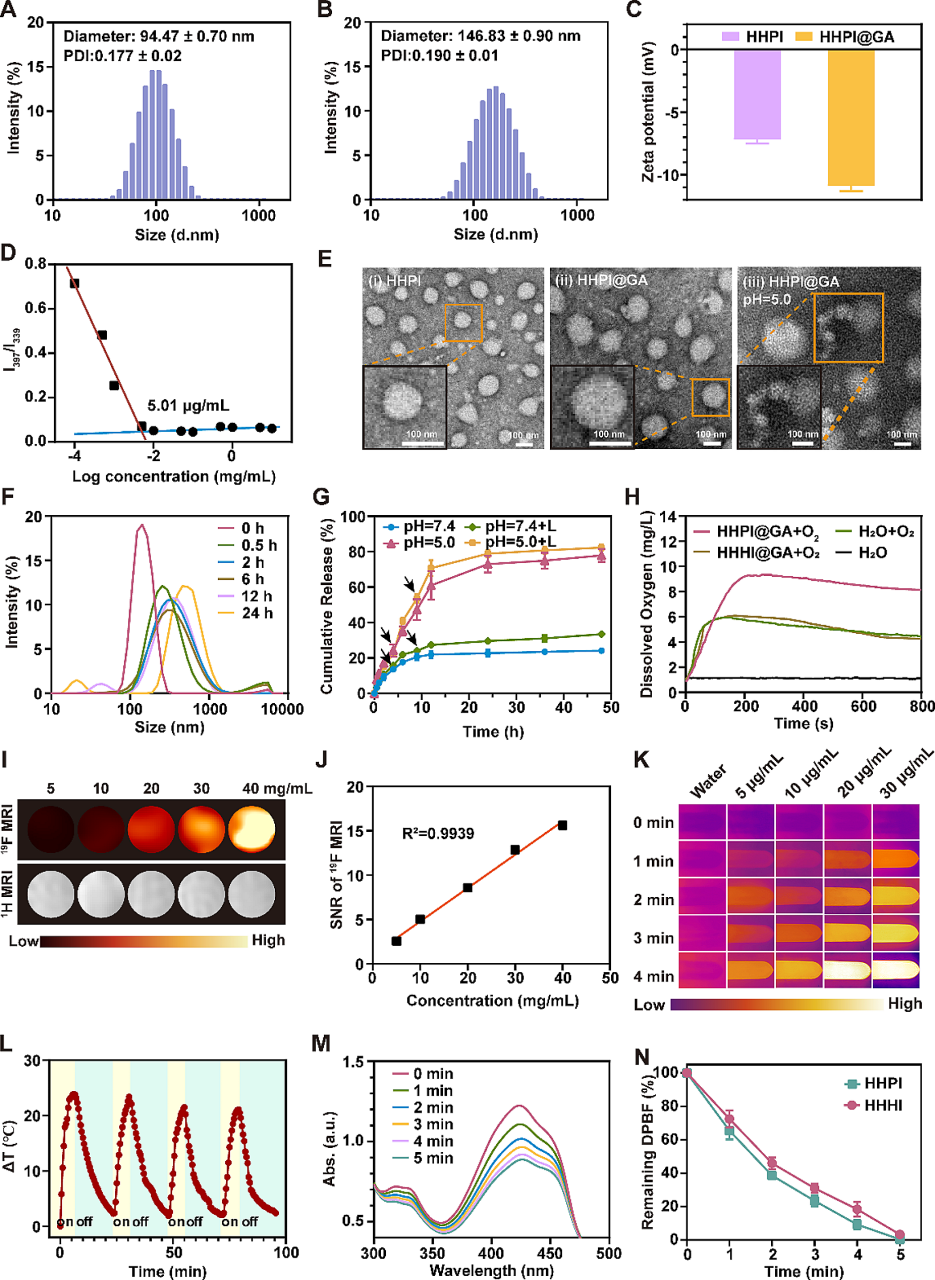
**A** Size distribution of HHPI by DLS. **B** Size distribution of HHPI@GA by DLS. **C** Zeta potentials of HHPI and HHPI@GA. **D **The critical micelle concentration (CMC) of HHPI@GA NPs. **E** TEM images of HHPI **(i)**, HHPI@GA **(ii)** and HHPI@GA at pH 5.0 **(iii)**. Scale bar: 100 nm.  **F** Size distribution of HHPI@GA at pH 5.0 from 0–24 h by DLS. **G** Release profiles of GA under various pH conditions (pH 5.0 and 7.4), with or without 808 nm laser irradiation. Black arrows indicate the points of laser irradiation, which occur at the 3rd and 9th hour marks, respectively. **H** Dissolved oxygen concentration in deoxygenated pure water after addition of oxygen-saturated HHHI@GA NPs and HHPI@GA NPs at different times. **I**^1^H MRI and ^19^F MRI images of HHPI@GA NPs at different concentrations. **J** Relationship between ^19^F MRI SNR and HHPI@GA NPs concentration. **K** IR thermal images of HHPI@GA NPs under laser irradiation. **L** The photothermal capability of HHPI@GA NPs over four laser on/off cycles. **M** The absorption spectra of DPBF upon laser irradiation in HHPI@GA NPs solution. **N** Normalized absorbance of DPBF at 420 nm during ROS detection after different treatments

DLS analysis indicated that the average diameters of the HHPI and HHPI@GA NPs were 94.47 ± 0.70 and 146.83 ± 0.90 nm, respectively, with a PDI of 0.177 ± 0.02 and 0.190 ± 0.01, and both samples were monodispersed **(**Fig. 1A-B**)**. The larger size of the HHPI@GA NPs compared to their drug-free counterparts (HHPI) was attributed to the encapsulation of GA. Zeta potential measurements indicated that both the HHPI and HHPI@GA surfaces were negatively charged **(**Fig. 1C**)**, which prevented the adsorption of plasma proteins and prolonged blood circulation [44]. Moreover, the quantitative micellization detected by CMC indicated the stability of the amphiphilic NPs. In Fig. 1D, the CMC of HHPI@GA was 5.01 µg/mL, which was low enough to maintain excellent cyclic thermodynamic stability in diluted conditions such as blood circulation before reaching tumor sites. Similarly, HHPI@GA demonstrated stability over 3 d in water, PBS, culture medium, and 10% serum, indicating its suitability for theragnostic applications **(Fig. S12D)**.

The TEM images revealed that both HHPI and HHPI@GA NPs exhibited clear uniform spherical shapes at pH 7.4. The particle size of HHPI@GA increased at pH 5.0, with expansion and extension, indicating that the pH sensitivity of HHPI NPs was induced by the protonation of H_6_**(**Fig. 1E**)**. The pH responsiveness of HHPI@GA was further evaluated. In PBS at pH 5.0, the average diameter of HHPI@GA exhibited two peaks, suggesting that the imidazole groups on the H_6_ group of HHPI absorbed a large number of protons at this pH, leading to an increase in size and accelerated drug release from the NPs **(**Fig. 1F**)**. This was corroborated by the in vitro drug release results for HHPI@GA at pH 5.0, which showed a higher rate and amount of drug release under acidic conditions than under neutral conditions **(**Fig. 1G**)**. Additionally, laser irradiation slightly enhanced the drug release at pH 7.4 and pH 5.0; however, the enhancement was subtle. It is hypothesized that the primary mechanism of drug release from HHPI@PA is attributed to the pH-responsive nature of polyhistidine, with the modest increase potentially arising from accelerated molecular dynamics induced by the photothermal effect [45].

To validate the oxygen-supplying function of PFC in HHPI@GA, He was used as a negative control to create He-containing NPs (HHHI@GA). As depicted in Fig. 1H, HHPI@GA exhibited a significant increase in dissolved oxygen compared with water and HHHI@GA. This indicated that HHPI@GA can act as an oxygen reservoir to alleviate hypoxia and increase local oxygen content.

Previous experiments have shown that PFC offer a strong ^19^F MR signal [40, 46]. The ^19^F NMR spectrum of HHPI@GA exhibited a strong singlet at − 74.5 ppm **(Fig. S12E)**. To further explore the potential of HHPI@GA in ^19^F MRI, imaging was performed on solutions with varying concentrations of HHPI@GA, and the signal-to-noise ratio (SNR) in the images was measured. As shown in Fig. 1I, distinct ^19^F MR ‘hot spot’ images were detectable, with the intensity of the ^19^F MR signals increasing with the concentration of HHPI@GA. The measured SNR results demonstrated a strong linear correlation between the intensity of the ^19^F MRI signal and HHPI@GA concentration **(**Fig. 1J**)**.

### Photothermal and photodynamic properties

The photothermal and photodynamic properties of HHPI@GA were evaluated. HHPI@GA exhibited a temperature-elevation curve over time upon 808 nm laser irradiation. In addition, the increase in temperature was concentration-and laser power-dependent **(Fig. S12F-G)**. The temperature of HHPI@GA rapidly increased by approximately 23 °C after irradiation (1 W/cm^2^) within 4 min, which was sufficient to destroy hyperthermia-mediated cancer cells. The IR thermal images also validated the laser-induced photothermal heating of HHPI@GA **(**Fig. 1K**)**. The photothermal stability of HHPI@GA was assessed through four irradiation NIR-on/off cycles and two cycles 24 h apart; no obvious reduction in photostability was observed **(**Fig. 1L **and Fig. S12H)**. These results demonstrated that HHPI@GA can be used in PTT.

Subsequently, the ROS generation ability of HHPI@GA NPs was assessed under 808 nm irradiation using DPBF as a singlet oxygen indicator. As shown in Fig. 1M, a time-dependent decrease in the absorption peak at 420 nm was observed, indicating the strong ROS generation ability of HHPI@GA NPs triggered by 808 nm laser irradiation. The level of ROS production is related to local oxygen content and photodynamic efficiency. To validate the enhancement of the PDT effect of grafted PFC in HHPI@GA, we synthesized HHHI@GA with He instead of PFC and compared the ROS production of HHHI@GA and HHPI@GA. The results showed that the HHPI@GA group displayed higher PDT efficiency, demonstrating the effectiveness of the grafted PFC in enhancing PDT **(**Fig. 1N**)**.

### Intracellular localization and ROS detection

**Fig. 2:**
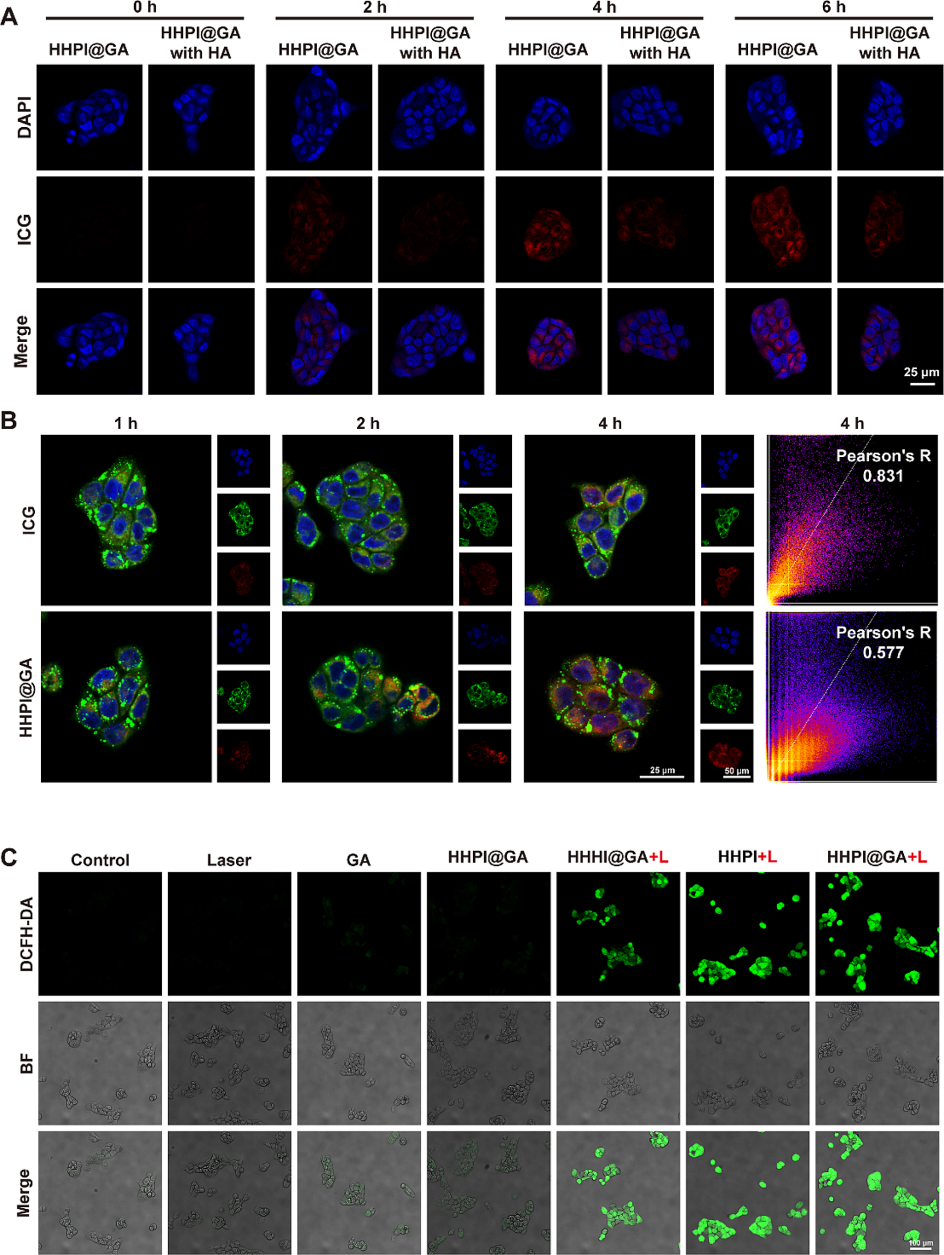
**A** Cell uptake CLSM images of HHPI@GA NPs in Cal-27 cells after incubation at 0 h, 2 h, 4 h, and 6 h with or without HA. Scale bar: 25 μm. **B** Lysosome escape CLSM images of free ICG and HHPI@GA NPs after incubation at 1 h, 2 h, and 4 h and Pearson correlation coefficient (Pearson’s R) for colocalization analysis of images at 4 h. Scale bar: 25 and 50 μm. **C** ROS generation in vitro CLSM images after different treatments of Cal-27 cells. Scale bar: 100 μm

CLSM was used to record the cellular uptake of HHPI@GA NPs in Cal-27 cells, with ICG in HHPI@GA serving as the fluorescent reference signal. As shown in Fig. 2A, significant fluorescence was observed in the cells within 4 h, indicating effective internalization of HHPI@GA. Furthermore, we investigated the influence of HA on the cellular uptake. The CD44 receptor is overexpressed on the surface of various cancer cells and binds to HA-modified carriers, which facilitates the active targeting of nanomedicines to cancer cells through ligand-receptor binding [47–49]. In this study, after free HA pretreatment, the fluorescence intensity was significantly reduced owing to the binding between free HA and CD44 receptors on Cal-27 cells. This observation was also corroborated by flow cytometry analyses, which similarly reflected a reduction in cell uptake. These findings indicate that HA-mediated active targeting is a crucial factor in the cellular uptake of HHPI@GA **(**Fig. 2A **and Fig. S13A)**.

Lysosome is a significant obstacle to drug delivery, and efficient lysosome escape ability is an important property required for advanced drug delivery systems. To study the lysosomal escape ability of HHPI@GA, the lysosomes of Cal-27 cells were labelled with LysoTracker Green and observed using CLSM after 1, 2, and 4 h of incubation. As illustrated in Fig. 2B **and Fig. S13B**, cells incubated with free ICG displayed yellow fluorescence in the cytoplasm (originating from the colocalization of red and green fluorescence), maintaining a high degree of colocalization (Pearson’s *R* = 0.831) after 4 h. This indicated that most of the ICG was trapped in the lysosome. In the HHPI@GA group, most of the red fluorescence overlapped with the green fluorescence after 2 h of incubation. However, at 4 h, red fluorescence was clearly observed outside the lysosome with a lower degree of colocalization (Pearson’s *R* = 0.577), demonstrating the lysosomal escape ability of HHPI@GA. Specifically, the acidic lysosome environment triggered the protonation of histidine, leading to ‘proton sponge’ effect, the osmotic pressure in lysosome increased and caused the burst of lysosome membrane, achieving the escape of HHPI@GA [50].

To verify the ROS generation ability of HHPI@GA, we measured intracellular ROS levels using the ROS probe DCFH-DA in Cal-27 and 4T1 cells, where green fluorescence indicated intracellular ROS levels. CLSM revealed that after 5 min of irradiation, the sequence of DCFH-DA fluorescence intensity was control, laser < GA < HHPI@GA < HHHI@GA + L < HHPI + L < HHPI@GA + L, consistent for both Cal-27 and 4T1 cells **(**Fig. 2C **and Fig. S13C and Fig. S14A-B)**. This confirmed that HHPI@GA could induce high levels of intracellular ROS generation under 808 nm laser irradiation. In addition, the fluorescence intensity of the HHPI@GA + L group was higher than that of the HHHI@GA + L group, which indicated that oxygen can be effectively transported to oxygen-depleted cancer cells through HHPI@GA, providing the foundation for enhanced PDT.

### In vitro anticancer evaluation

**Fig. 3:**
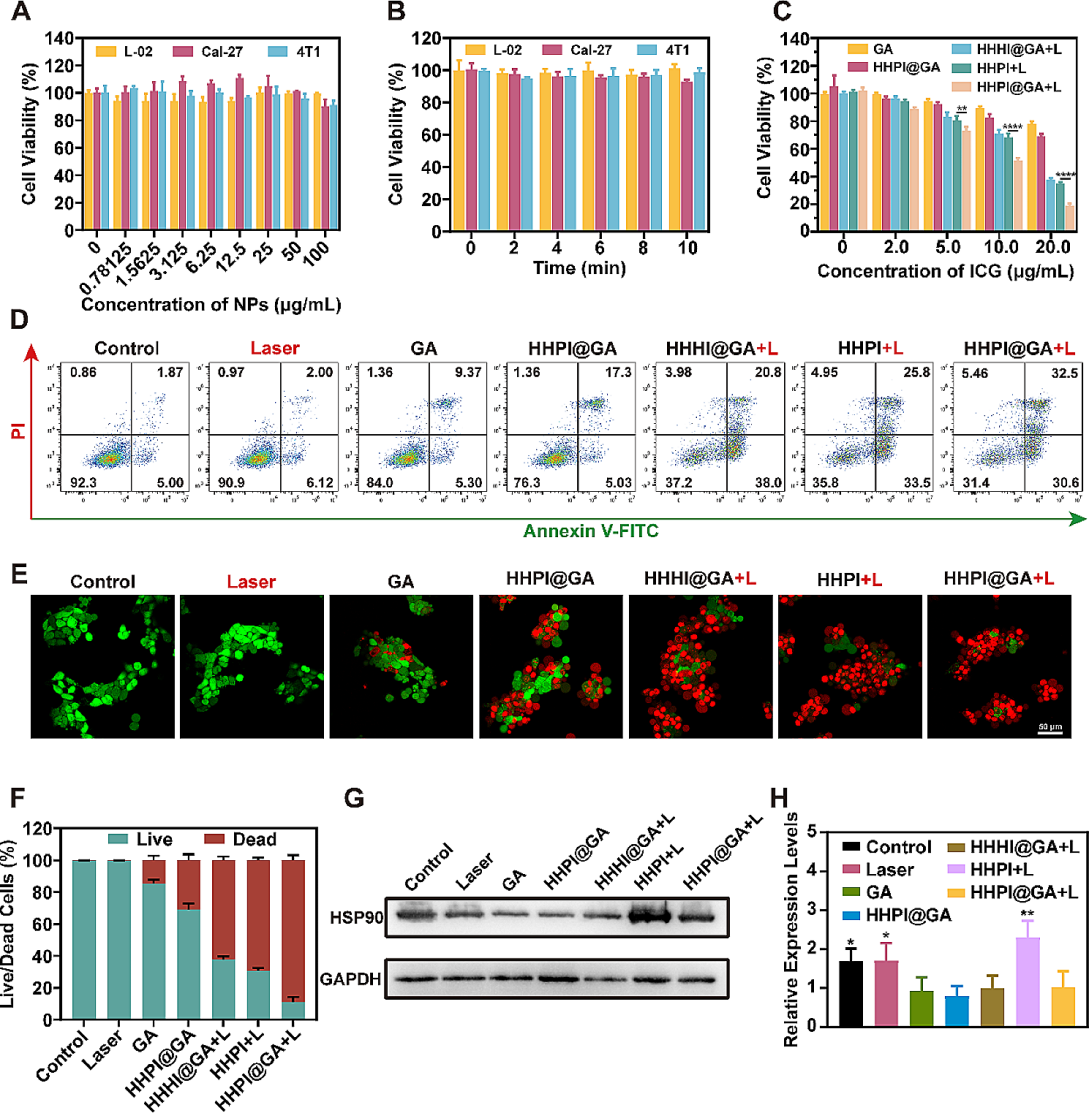
**A** Cell viability of L-02, Cal-27, and 4T1 cells incubated with different concentrations of blank HHPI@GA NPs for 24 h measured by CCK-8 assay. **B** Cell viability of L-02, Cal-27, and 4T1 cells after 0–10 min laser irradiation. **C** Cell viability of Cal-27 cells after various treatments. **D** Apoptosis analysis of Cal-27 cells after various treatments. **E, F** CLSM images **(E)** and semi-quantitative analysis **(F)** of live/dead cell assays of Cal-27 cells. Scale bar: 50 μm. **G, H** Western blot of HSP90 expression in Cal-27 cells with different treatments **(G)** and corresponding quantitative analysis (HHPI@GA + L vs. other groups) **(H)**. Data are shown as mean ± SD (*n* = 3) (*P < 0.05, **P < 0.01, ***P < 0.001, and ****P < 0.0001)

The CCK-8 assay was used to assess the biocompatibility of the 808 nm laser irradiation and blank HHPI NPs solution. As shown in Fig. 3A-B, cells treated with 808 nm laser irradiation for 0–10 min or with HHPI at concentrations ranging from 0 to100 µg/mL maintained a viability above 90%. This indicated that neither laser irradiation alone nor blank HHPI NPs exhibited significant cytotoxicity towards the cells. In addition, as shown in **Fig. S14C**, even the highest concentration of blank HHPI NPs did not induce hemolysis, indicating good blood compatibility.

To evaluate the in vitro anticancer efficacy of the HHPI@GA NPs against Cal-27 and 4T1 cells, we first assessed the cytotoxicity in different groups using the CCK-8 assay **(**Fig. 3C **and Fig. S14D)**. The results indicated that HHPI@GA NPs exhibited the most pronounced cytotoxicity under 808 nm laser irradiation. The difference between the HHHI@GA + L and HHPI@GA + L groups was attributed to the enhanced PDT effect of PFC, whereas the difference between the HHPI + L and HHPI@GA + L groups was attributed to the enhanced PTT effect caused by GA (which inhibited the expression of HSP90) [51]. The cytotoxicity test showed that enhanced PTT and PDT strategy effectively improved the lethality toward cancer cells. Further assessment was conducted through the Annexin V-FITC/PI assays to analyze cell apoptosis levels using flow cytometry. High levels of cell apoptosis were observed in the HHHI@GA + L, HHPI + L, and HHPI@GA + L groups, with HHPI@GA + L inducing the most significant apoptosis **(**Fig. 3D **and Fig. S15A)**. Subsequently, live/dead cell staining assay was performed. Calcein-AM staining (green) indicated live cells, and PI staining (red) indicated dead cells. The CLSM images of live and dead cells were consistent with those of the CCK-8 and Annexin V-FITC/PI assays, which further confirmed the anticancer activity of HHPI@GA NPs **(**Fig. 3E-F **and Fig. S15B-C)**.

Hyperthermia caused by PTT can effectively kill cancer cells; however, after a certain time, cancer cells overexpress HSPs to protect themselves from heat damage and resist PTT, which affects the inhibitory effect of PTT [52]. Recent studies have shown that GA has a highly effective inhibitory effect on HSP90 expression, thereby restoring the sensitivity of cells to heat [15, 51]. To investigate the anticancer mechanism of HHPI@GA, Western blotting was performed to analyze the expression of HSP90 in Cal-27 cells subjected to different treatments **(**Fig. 3G-H**)**. The Western blotting results indicated that cells in group HHPI + L showed increased expression of HSP90 owing to the PTT effect, whereas in the presence of GA, the expression level of HSP90 was significantly downregulated. This suggests that HHPI@GA efficiently inhibited the expression of HSP90 in cancer cells induced by PTT, thereby enhancing the therapeutic efficacy of PTT.

### In vivo fluorescence and ^19^F MRI

The diagnostic imaging task serves as a foundational step in assessing disease severity and facilitating accurate evaluation. Fluorescence and magnetic resonance (MR) imaging are distinguished as predominant techniques for the early detection of cancer and its metastases, attributed to their comprehensive imaging capabilities [28]. In our research, we selected oral cancer and breast cancer cell lines to construct the primary cancer models. It is well-acknowledged that lung metastasis represents a prevalent progression in both oral and breast cancer, markedly influencing patient prognosis and treatment approaches [25]. Consequently, we opted for the lung metastasis model to epitomize the metastatic tumor paradigm. The presence of ICG and PFC in HHPI@GA enabled in vivo dual-modality imaging with ICG-mediated fluorescence and PFC-mediated ^19^F MRI, offering a novel strategy for the diagnosis of primary and metastatic cancers.

### Tumor-bearing mouse model

**Fig. 4:**
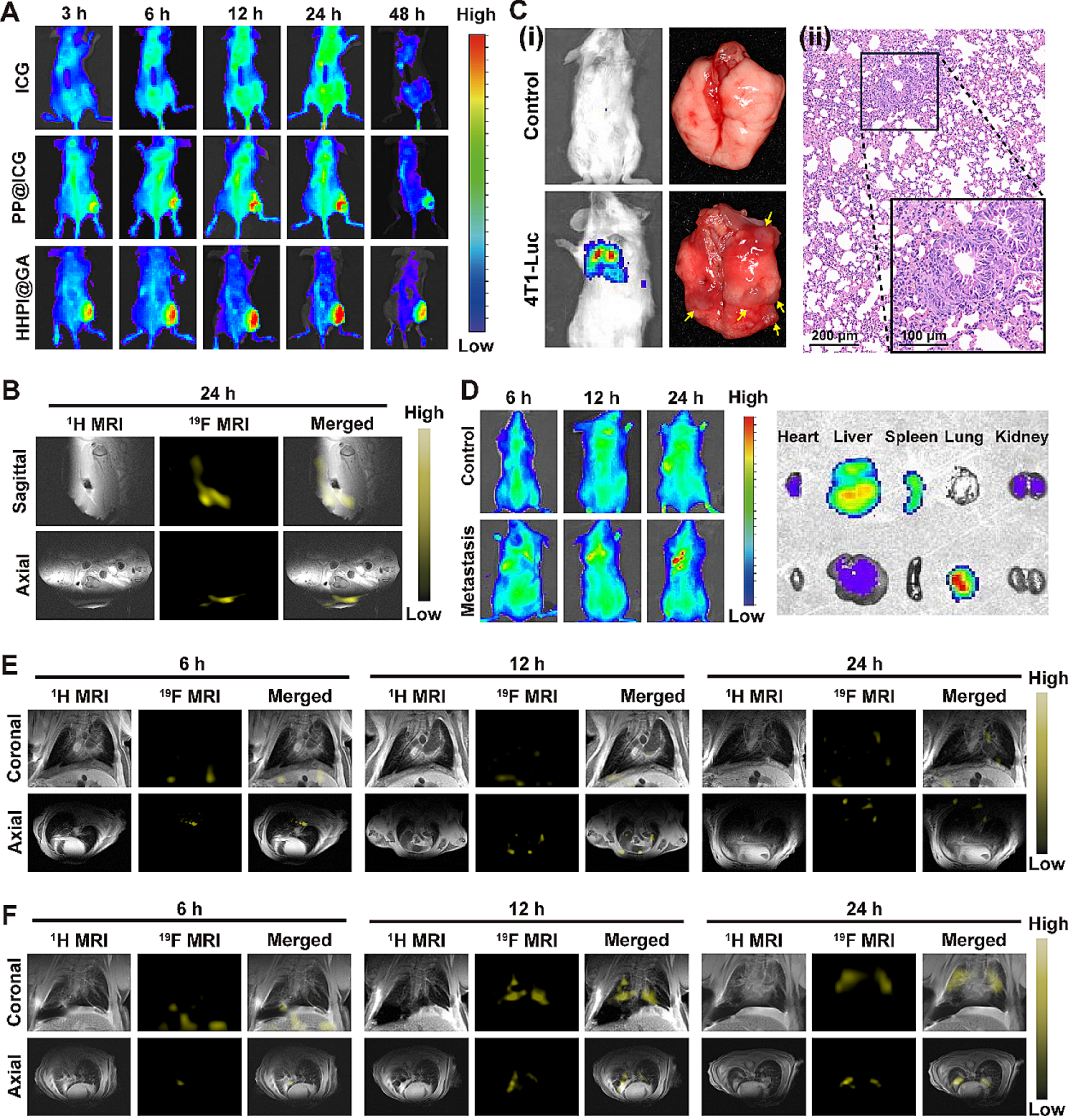
**A **In vivo fluorescence images of Cal-27 tumor-bearing mice at 3, 6, 12, 24, and 48 h after intravenous injection of free ICG, PP@ICG and HHPI@GA NPs. **B** Sagittal and axial MRI of the tumor-bearing mice after intravenous injection of HHPI@GA NPs at 24 h. The ^1^H MRI are overlaid with the ^19^F images. **C (i)**In vivo bioluminescence images and ex vivo lung photographs of untreated and lung metastasis mice after injection of luciferin potassium salt solution. Yellow arrows represented the foci of cancer metastases. **(ii)** The histological analyses of lung sections of lung metastasis mice by H&E staining. Scale bar: 200 μm (100 μm inset). **D **In vivo fluorescence images of untreated and lung metastasis mice at 6, 12, and 24 h after intravenous injection of HHPI@GA NPs, and ex vivo fluorescence images of major organs and tumor at 24 h. **E, F** Coronal and axial MRI of the untreated **(E)** and lung metastasis mice **(F)** after intravenous injection of HHPI@GA NPs at 6, 12 and 24 h. The ^1^H MRI are overlaid with the ^19^F images

Primary cancers were simulated using a subcutaneous tumor model. The biodistribution of HHPI@GA in Cal-27 tumor-bearing mouse model was assessed using in vivo and ex vivo organ fluorescence imaging. As depicted in Fig. 4A **and Fig. S16A**, owing to the EPR effect, both mPEG-PCL@ICG (PP@ICG) and HHPI@GA accumulated in the tumors at 3 h post-injection. Over time, the HHPI@GA signal gradually increased in tumors, significantly surpassing the fluorescence intensity of both the PP@ICG and ICG groups, peaking at 24 h post-injection. This was attributed to the dual capabilities of HHPI@GA for passive and HA-mediated active targeting. Furthermore, the HHPI@GA group maintained substantial signal intensity at 48 h, whereas the signals from the PP@ICG and ICG groups were diminished, indicating that HHPI@GA continuously accumulated in the tumor. Ex vivo fluorescence images of tumors excised 48 h after intravenous administration revealed that the fluorescence intensity within the tumors treated with HHPI@GA was substantially higher than those in the other two groups **(Fig. S16B)**, further confirming the superior tumor-targeting and accumulation properties of HHPI@GA. The ^19^F MRI results at 24 h also corroborated these findings.

In vivo soft tissues lack endogenous ^19^F signals, thus endowing ^19^F MRI with minimal background noise and high sensitivity [46, 53]. In addition, ^19^F MRI offers deep penetration and high resolution [54]. As illustrated in Fig. 4B, a pronounced ^19^F MRI ‘hot spot’ signal was visible at the orthotopic tumor site 24 h after the tail vein injection of HHPI@GA, which was consistent with the results of in vivo fluorescence imaging, demonstrating the dual-modality imaging capabilities of HHPI@GA in primary cancers.

### Lung metastasis mouse model

Metastatic cancers were simulated in a lung metastasis mouse model. 4T1-luc cells were chosen because of their easy metastatic and bioluminescence characteristics [55, 56]. 4T1-luc mice model of lung metastasis was established, while untreated healthy mice were used as the control. Using bioluminescence imaging based on 4T1-luc cells, and H&E staining of lung tissues, we confirmed the successful establishment of lung metastasis mice model from both imaging and histopathological perspectives **(**Fig. 4C**)**. The biodistribution of HHPI@GA in the lung metastasis mice model were initially assessed using both in vivo and ex vivo organ fluorescence imaging. As shown in Fig. 4D, the fluorescence intensity in the lungs of the metastatic group gradually increased and reached its peak at 24 h post-injection, whereas the fluorescence of the healthy group was mainly distributed in the liver and spleen. This indicated that HHPI@GA effectively accumulated in lung metastatic lesions, thereby aiding in the diagnosis of cancer metastasis. Furthermore, the in vivo^19^F MRI capability of HHPI@GA for metastatic cancer imaging was evaluated using a 9.4 T MRI. As shown in **Fig. 4E-F**, ^19^F MRI signals were detectable in the lungs and liver of mice at 6 h post-injection. Over time, the ^19^F MRI signals progressively accumulated in the lungs, with strong ‘hot spot’ signals evident at 24 h, consistent with the fluorescence imaging results, demonstrating the dual-modality imaging capabilities of HHPI@GA in metastatic cancers.

These results indicated that HHPI@GA can simultaneously achieve ICG-mediated fluorescence imaging and PFC-mediated ^19^F MRI in primary and metastatic cancers. This dual-modality imaging capability endowed HHPI@GA with a significant diagnostic potential for clinical cancer treatment.

### In vivo anticancer efficiency

**Fig. 5:**
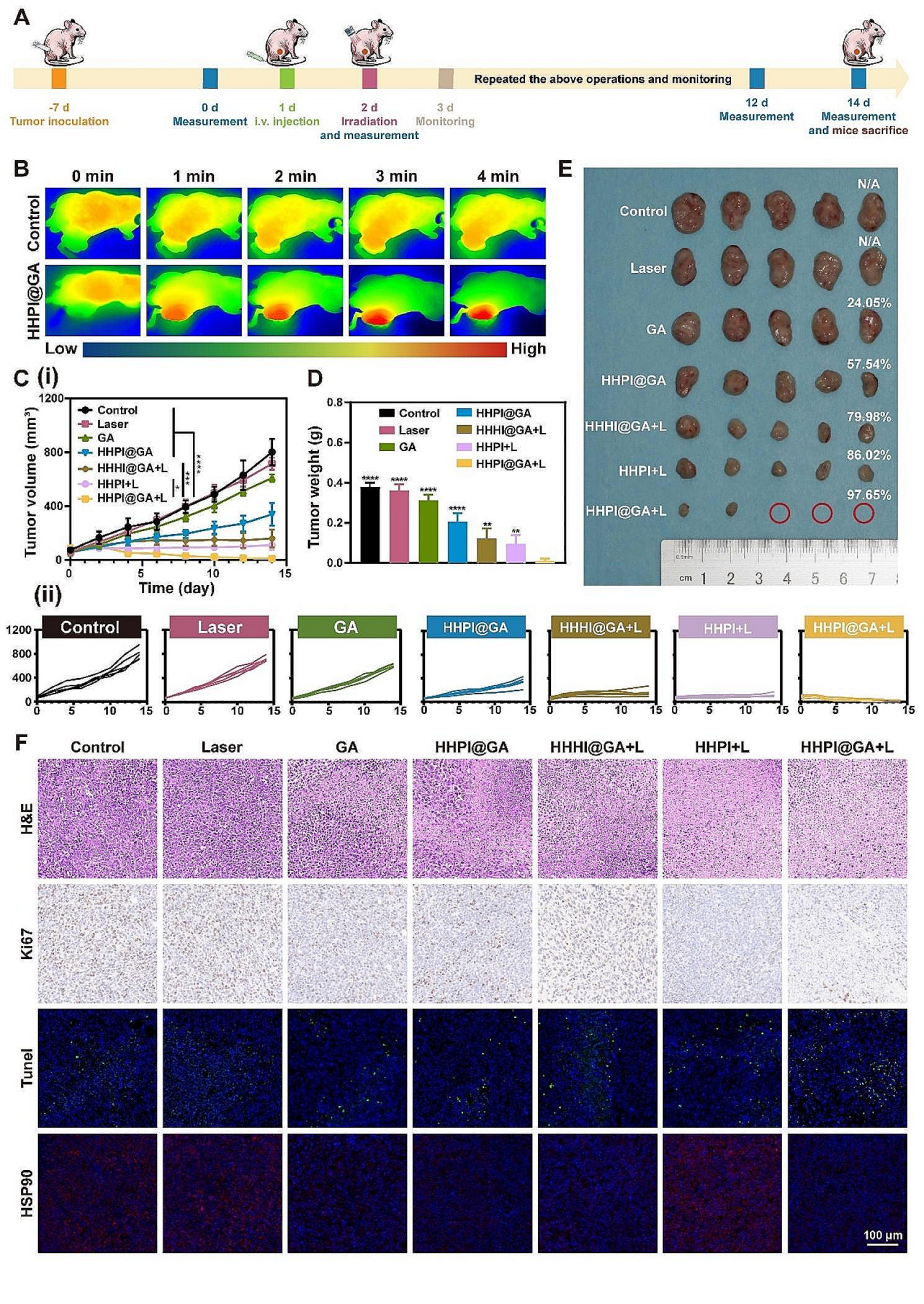
**A** Schematic of treatments in Cal-27 tumor-bearing mice. **B** Near-infrared thermography of mice with different treatments under laser irradiation. **C** Tumor volume **(i)** and tumor growth curves **(ii)** of Cal-27 tumor-bearing mice. **D** Tumor weights of different treatment groups in Cal-27 tumor-bearing mice. **E** Photographs and tumor inhibition ratio (TIR) values of tumors after 14-day treatments. **F **Ex vivo histological analyses of tumor sections after various treatments by H&E, Ki67, TUNEL and HSP90 immunofluorescence staining. Scale bar: 100 μm. Data are shown as mean ± SD (*n* = 5) (*P < 0.05, ***P < 0.001, and ****P < 0.0001)

Cal-27 and 4T1 tumor-bearing mouse models were used to evaluate the in vivo anticancer efficacy of HHPI@GA **(**Fig. 5A **and Fig. S17A)**. Treatments were initiated when the tumors reached a volume of 50–100 mm^3^. During the treatment, thermal imaging was used to monitor temperature changes at tumor sites in Cal-27 tumor-bearing mice following the injection of HHPI@GA. Based on the results of the in vivo distribution study, the tumor sites were irradiated with an 808 nm laser at 24 h post-injection, coinciding with the peak accumulation of NPs in tumors, to conduct localized PTT. The results revealed a rapid increase in the tumor temperature to over 45 °C within 4 min. In contrast, only a slight temperature rise was observed in the control group. The significant temperature change at tumor sites indicated that HHPI@GA could effectively accumulate in tumor areas, achieving desired PTT effects (Fig. 5B).

Mouse weight and tumor volume were recorded every other day for 14 days for Cal-27 tumor-bearing mice and 12 days for 4T1 tumor-bearing mice. The results of tumor volume changes indicated that the tumor volumes in both the control and laser-only groups increased rapidly, while the remaining five groups exhibited varying degrees of tumor suppression **(**Fig. 5C **and Fig. S17B)**. Compared with the treatment groups without laser irradiation, those subjected to laser irradiation exhibited significantly enhanced tumor suppression, which was attributed to the excellent PTT and PDT effects of ICG. The enhanced PTT and PDT effects of HHPI@GA resulted in superior in vivo anticancer effects, ultimately achieving exceptional tumor inhibition rates (TIRs) of over 95% in both Cal-27 and 4T1 tumor-bearing mice. The difference between the HHHI@GA + L and HHPI@GA + L groups was attributed to the enhanced PDT effect of grafted PFC, while the difference between the HHPI + L and HHPI@GA + L groups was due to the enhanced PTT effect caused by GA, which inhibited the expression of HSP90. Mice treated with Cal-27 and 4T1 cells were euthanized to harvest tumors and major organs at 14 and 12 d post-treatment, respectively, and their tumor weights were measured **(**Fig. 5D-E **and Fig. S17C-D)**. Throughout the treatment period, all the mice were monitored for body weight changes, and no significant weight loss was observed, indicating the relatively high biosafety of HHPI@GA **(Fig. S17E-F)**.

As illustrated in Fig. 5F, further histological and immunohistochemical analyses were conducted on tumors from the seven mouse groups. The results of H&E staining and the Ki67 and TUNEL assays demonstrated great anticancer effects in the HHHI@GA + L, HHPI + L, and HHPI@GA + L groups. Immunohistochemical results for HSP90 indicated that HHPI@GA significantly inhibited the expression of HSP90 in cancer cells, thereby restoring the thermal sensitivity of the tumors to PTT and enhancing the therapeutic effect. In addition, H&E staining was performed on the major organs of mice to monitor the potential toxicity of HHPI@GA *in vivo.* No significant morphological changes were observed in the HHPI@GA group, confirming its good biocompatibility from a histological perspective **(Fig. S18)**.

## Conclusion

In summary, we successfully developed a dual-modality imaging nanoplatform (HHPI@GA) for enhanced phototherapy of primary cancer and the detection of cancer metastasis. After intravenous injection, HHPI@GA NPs accumulated in tumor tissues via EPR effect and HA-mediated active targeting, releasing oxygen and regulating local hypoxic conditions. Meanwhile, GA was released due to the acidic environment and then inhibited the expression of HSP90, resulting in augmented phototherapy therapy under NIR laser irradiation. In addition, ICG and PFC enriched in tumor tissues were used as fluorescent and ^19^F MRI probes, respectively, to indicate cancer metastasis, realizing dual-modality imaging. Overall, this nanoplatform overcame key challenges in conventional phototherapy for cancer treatment, offering a promising strategy for efficient cancer metastasis diagnosis, and displaying broad clinical application prospects.

## Experimental section

### Materials

Hyaluronic acid-Thiol (HA-SH) (Mw = 10000 g/mol) was obtained from Guangzhou Weihua Biotechnology Co., Ltd. (Guangzhou, China). GL Biochem Ltd. (Shanghai, China) was the source of CH_6_K(Fmoc) (CHHHHHHK(Fmoc)-CONH_2_) (Mw = 1293 Da). ICG-COOH (Mw = 609 Da) and mPEG-PCL (PP) (Mw = 10000 Da) was procured from Xi’an ruixi Biological Technology Co., Ltd. (Xi’an, China). Gambogic acid was purchased from Chengdu Desite Biotechnology Co., Ltd. (Chengdu, China). 1H,1H-Undecafluorohexylamine (PFC-NH_2_) was acquired from Zhengzhou Alfa Chemical Co., Ltd. (Zhengzhou, China). 1-Hexadecylamine (He-NH_2_), 3-MaleimDaidopropionic acid (Mal-COOH), O-benzotriazole-N, N, N’, N’-tetramethyluronium hexafluorophosphate (HATU), N, N-diisopropylethylamine (DIPEA), N-hydroxysuccinimide (NHS), N-(3-dimethylaminopropyl)-N′-ethylcarbondiimine hydrochloride (EDC·HCl), D-Luciferin potassium salt and anhydrous N, N-dimethylformamide (DMF) were purchased from Adamas Reagent, Ltd. (Shanghai, China).

Dulbecco’s modified Eagle’s medium (DMEM), RPMI 1640 medium and Fetal bovine serum (FBS) were procured from Thermo Fisher Scientific Inc. (Massachusetts, USA). Hoechst 33342, LysoTracker Green DNA-26, 2’,7’-dichlorodihydrofluorescein diacetate (DCFH-DA), 1,3-diphenylisobenzofuran (DPBF), Calcein AM/PI assay kit, Cell counting kit-8 (CCK-8), and Annexin V-FITC/PI were obtained from Dalian Meilun Biotechnology Co., Ltd. (Dalian, China). Anti-Hsp90 Rabbit pAb and glyceraldehyde-3-phosphate dehydrogenase (GAPDH) were purchased from Wuhan Servicebio Technology Co., Ltd. (Wuhan, China).

L-02 cells (human liver normal cells), Cal-27 cells (human tongue squamous cell carcinoma cells) and 4T1 cells (mouse breast cancer cells) were purchased from the Cell Bank of the Chinese Academy of Sciences (Shanghai, China). 4T1-luc cells were purchased from Guangzhou Yuanjing Biotechnology Co., Ltd. (Guangzhou, China). Male BALB/c nude mice and female BALB/c mice were provided by Beijing HFK Bioscience Co. Ltd. (Beijing, China).

### Synthesis of PFC-Mal

Initially, Mal-COOH (169.57 mg, 1.0 mmol), NHS (576.92 mg, 5.0 mmol), and EDC·HCl (1916.39 mg, 10.0 mmol) were dissolved in 15 mL of anhydrous DMF and stirred at 25 °C for 2 h to activate the carboxyl groups of Mal-COOH. Subsequently, PFC-NH_2_ (150 mg, 0.5 mmol) dissolved in 10 mL DMF was added dropwise to the activated Mal-COOH solution. Further stirring at 25 °C for 1 d under nitrogen atmosphere completed the amidation process. The reaction product was subsequently placed in a dialysis tube (MWCO = 1000 Da) and dialyzed against DI water for 1 d to eliminate residual reactants. Finally, PFC-Mal was obtained by lyophilization.

### Synthesis of H_6_-PFC

PFC-Mal (144.67 mg, 0.32 mmol) and CH_6_K(Fmoc) (208 mg, 0.16 mmol) were dissolved in 15 mL DMF. The formation of PFC-CH_6_K(Fmoc)(H_6_-PFC) ensued upon stirring the mixture at 25 °C for 1 d under a nitrogen atmosphere. Subsequently, pure H_6_-PFC was isolated by dialysis (MWCO = 1000 Da) against DMF and DI water over 2 d, followed by lyophilization.

### Synthesis of H_6_-PFC-ICG

Initially, ICG-COOH (181.58 mg, 0.3 mmol), HATU (113.3 mg, 0.3 mmol), and DIPEA (103.67 µL, 0.6 mmol) were dissolved in 15 mL anhydrous DMF. The solution was then stirred at 25 °C for 30 min to activate the carboxyl groups of ICG-COOH. Subsequently, H_6_-PFC (260 mg, 0.15 mmol) was fully dissolved in 10 mL anhydrous DMF and added dropwise to the activated ICG-COOH solution. Amidation ensued upon agitating the mixture for 30 min at 25 °C under a nitrogen atmosphere. Finally, pure H_6_-PFC-ICG was isolated by dialysis (MWCO = 1000 Da) against DMF and DI water for 2 d, followed by lyophilization.

### Synthesis of NH_2_-H_6_-PFC-ICG

The Fmoc groups of the synthesized H_6_-PFC-ICG were deprotected by treating H_6_-PFC-ICG with 20% piperidine/DMF (2 × 5 min). This solution was then added dropwise to cold diethyl ether and ultrasonicated to ensure complete dissolution. The mixture was then centrifuged at 10,000 rpm for 5 min, and the supernatant was discarded. The precipitate was washed twice with diethyl ether. The final precipitate was dried to obtain pure NH_2_-H_6_-PFC-ICG.

### Synthesis of Mal-H_6_-PFC-ICG

Initially, Mal-COOH (44.78 mg, 0.26 mmol), HATU (100.7 mg, 0.26 mmol), and DIPEA (192.15 µL, 0.52 mmol) were dissolved in 10 mL anhydrous DMF, followed by stirring at 25 °C for 30 min to activate the carboxyl groups of Mal-COOH. Subsequently, NH_2_-H_6_-PFC-ICG (280 mg, 0.13 mmol) was fully dissolved in 10 mL DMF and added dropwise to the activated Mal-COOH solution. Amidation proceeded upon stirring the mixture for 30 min at 25 °C under a nitrogen atmosphere. Finally, pure Mal-H_6_-PFC-ICG was isolated by dialysis (MWCO = 1000 Da) against DMF and DI water for 2 d, followed by lyophilization.

### Synthesis of HHPI

Initially, HA (150 mg, 0.015 mmol) was dissolved in 10 mL DI water. Mal-H_6_-PFC-ICG (271.68 mg, 0.12 mmol) powder was dissolved in 10 mL DMF and added dropwise to the HA solution and stirred for 24 h. The resulting mixture was dialyzed (MWCO = 10,000 Da) against DMF and DI water for 2 d to yield pure HHPI.

### Preparation of HHPI@GA

GA (0.5 mg) and HHPI (10 mg) were ultrasonicated in 1 mL DMF until they were completely dissolved. This solution was added dropwise to 15 mL DI water and stirred for 24 h. Finally, the resulting mixture was dialyzed (MWCO = 3500 Da) against DI water for 2 d to obtain HHPI@GA.

HA-H_6_-He-ICG (HHHI) and HHHI@GA were prepared using a similar approach, with PFC-Mal replaced with He-Mal during the synthesis process.

### Characterization

The molecular weights of HHPI, HHHI, and their intermediate products were determined using electrospray ionization mass spectrometry (LCQ Orbitrap XL, USA). Gel permeation chromatography (GPC, Agilent 1200, USA) was used to determine the outflow times of HA, HPI, HHPI, HHI, and HHHI. Fourier-transform infrared spectroscopy (FTIR; Nicolet 6700, USA) was used to obtain the spectra of HA, HPI, HHPI, HHI, and HHHI to further verify the synthesis of HHPI and HHHI. The size distribution, polydispersity index (PDI), zeta potential, and morphology of HHPI and HHPI@GA NPs were characterized using dynamic light scattering (DLS; Nano ZS90, UK) and transmission electron microscopy (TEM, JEM2010, Japan).

### Drug‑loading capacity and encapsulation efficiency of HHPI@GA

The GA encapsulated within HHPI@GA was collected using the methanol demulsification method and quantified using a UV-vis-NIR spectrometer (PerkinElmer LAMBDA 950, USA) based on the standard curve of free GA. The absorbance of GA was measured at an excitation wavelength of 360 nm. The drug-loading capacity (DLC) and encapsulation efficiency (EE) were calculated using the following formulas: DLC (%) = [weight of loaded GA / (total weight of loaded GA and NPs)] × 100%; EE (%) = [weight of loaded GA / weight of feeding GA] × 100%.

### Critical micelle concentration (CMC)

Pyrene fluorescence analysis was performed to determine the critical micelle concentration (CMC) of HHPI@GA. Initially, a 1 × 10^− 4^ mg/mL pyrene/acetone solution was prepared and transferred into several brown volumetric flasks, left overnight to evaporate the acetone until dry. Subsequently, HHPI@GA solutions of varying concentrations were added to the volumetric flasks to achieve a final pyrene concentration of 2 × 10^− 6^ mg/mL. The solutions were consistently shaken at a constant temperature for 24 h. Utilizing a fluorescence spectrophotometer (F-7100, Japan), the excitation spectra of pyrene were detected, and the fluorescence emission intensities at 339 nm (*I*_*339*_) and 397 nm (*I*_*397*_) were measured. Finally, the CMC was calculated based on the *I*_*339*_ and *I*_*397*_ values.

### Stability and pH-responsive ability

The stability of the HHPI@GA NPs was assessed by incubating them in water, PBS, serum-free media, and PBS containing 10% serum at 37 °C, while their pH-sensitivity was assessed by incubating them in PBS at pH values of 7.4 and 5.0 at 37 °C. Size distribution measurements were performed at various time points using DLS and TEM.

### Hemolysis test

Hemolysis assays were conducted to evaluate the biosafety of HHPI@GA before in vivo use. HHPI@GA solutions of various concentrations were added to 2% red blood cell (RBC) suspensions obtained from BALB/c mice. PBS and DI water were used as the negative and positive controls, respectively. After thorough mixing, the samples were incubated at 37 °C for 2 h, followed by centrifugation at 3500 rpm for 8 min. Photographs were taken and the supernatants were collected for analysis. The absorbance of the supernatant was measured at 570 nm using UV-Vis-NIR spectrophotometry. The hemolysis rate of the RBCs was calculated using the following formula:

Hemolysis = [(*A*_*sample*_ − *A*_*(−)*_)/(*A*_*(−)*_ − *A*_*(+)*_)] × 100%.

### Drug release in vitro

The in vitro release profiles of GA from HHPI@GA were accurately measured under sink conditions. Initially, 3 mL of the HHPI@GA nanoparticle solution was allocated into three dialysis bags (MWCO = 3500 Da), each submerged in 100 mL of PBS containing 0.1% Tween 80, adjusted to pH levels of 7.4 and 5.0, and maintained under constant agitation at 37 °C. Subsequently, the samples were subjected to 808 nm laser irradiation for 5 min at the 3rd and 9th hours across the different pH conditions. At specific intervals, 1 mL samples from the external buffer of the dialysis bags were collected to determine the GA concentration utilizing a UV-visible spectrometer. Simultaneously, a corresponding volume of fresh PBS was supplemented to keep the volume constant for further analysis.

### Photothermal effects

A range of HHPI@GA solutions at varying concentrations were exposed to 808 nm laser irradiation (1 W/cm^2^) for 420 s, and the temperature was recorded by a thermocouple thermometer every 30 s. Meanwhile, the PBS and HHPI@GA solution (30 ug/mL) were photographed by an IR thermal imager. In addition, the photothermal response of HHPI@GA under various laser power intensities (0.5, 1.0, and 2.0 W/cm^2^) was examined. The photothermal stability of HHPI@GA was assessed by subjecting it to an 808 nm laser irradiation (1.0 W/cm^2^) for 5 min (laser on), followed by a natural cooling period (laser off). This on/off cycle was repeated four times. Similarly, open and closed loops were first performed, and another loop was performed 24 h later. Subsequently, thermal imaging photographs were obtained using an IR thermal imager (FLIR E5-XT, USA), and the temperature changes were recorded.

### ROS detection in vitro

DPBF was used as a probe to assess ROS generation by the HHPI@GA solution under 808 nm laser irradiation. Initially, a defined volume of HHPI@GA was mixed with DPBF. This combination was then subjected to 808 nm laser irradiation to record the absorbance intensity at 420 nm in a UV-vis-NIR spectrometer at 1 min intervals. ROS production induced by HHPI@GA was quantified by monitoring the fluorescence intensity changes in DPBF at 420 nm.

### Oxygen release

An oxygen release experiment was conducted using a dissolved oxygen meter to ascertain the oxygen levels in various solutions (HHPI@GA, HHHI@GA, and water). A preoxygenated sample solution (10 mL) was added to 50 mL deoxygenated water. The oxygen content in the water was continually monitored and documented at 5 s intervals for a duration of 800 s using a dissolved oxygen meter.

### ^19^F MRI

^19^F Nuclear magnetic resonance (NMR) spectroscopy was performed by dissolving HHPI@GA in DMSO-*d*_*6*_. Spectra were acquired at 298 K using a Bruker Avance 400 MHz spectrometer (Germany). All chemical shifts (δ) are reported in ppm.

Imaging of phantoms with NP solutions was performed using a BioSpec 94/30 USR 9.4 T small animal MRI scanner (Bruker, Germany). The solutions were placed in 5 mm NMR tubes within a 40 mm ^1^H/^19^F dual resonator volume coil. For sample localization, ^1^H MR images were obtained using a rapid acquisition with relaxation enhancement (RARE) sequence (TE = 2.3 ms, TR = 100 ms, flip angle (FA) = 30°, slice interval (SI) = 1 mm, field of view (FOV) = 40 × 40 mm^2^, matrix = 256 × 256). ^19^F MR images were acquired in the same stereotactic space as the ^1^H MR images using the RARE sequence (TE = 4.62 ms, TR = 1000 ms, FA = 180°, SI = 1 mm, FOV = 30 × 30 mm^2^, matrix = 32 × 32, scan time = 13 min 20 s).

### Cellular uptake and endo-lysosomal escape of HHPI@GA

Cal-27 cells were seeded in various 35-mm glass plates at a density of 1 × 10^4^ cells per well and cultured overnight. Subsequently, half of the plates were pretreated with free HA for 2 h to saturate the CD44 receptors, while the other half remained untreated. HHPI@GA was introduced into the glass plates, followed by the incubation of the cells for 0, 2, 4, and 6 h, after which the culture medium was removed. After washing thrice with PBS, the cells were fixed with 4% paraformaldehyde for 15 min. The cell nuclei were stained with Hoechst 33,342 (excitation (Ex)/emission (Em): 346/460 nm, 5 min). After washing with PBS, Cal-27 cells were imaged using confocal laser scanning microscopy (CLSM).

For the flow cytometry analysis, Cal-27 cancer cells (1.5 × 10^6^) were cultured in 6-well plates and separated into two distinct groups. Following a 24-hour cultivation period and the saturation of the CD44 receptor in one of the groups, a culture medium infused with HHPI@GA nanoparticles was introduced for incubation periods of 2, 4, and 6 hours. Subsequently, cells from both groups were treated with trypsin, subjected to centrifugation, and rinsed with PBS. The fluorescence intensities of the Cal-27 cells from each group were then quantified after 2, 4, and 6 hours using a BD FACS Calibur flow cytometer (Becton Dickinson, USA).

The cells were seeded on glass plates using the method described above and cultured overnight. Subsequently, free ICG or HHPI@GA was added to two of the plates, followed by incubation for 1, 2, or 4 h. After fixing with 4% paraformaldehyde, the cells were stained with LysoTracker Green (Ex/Em: 504/511 nm, 15 min) and Hoechst 33,342 (5 min). Images of Cal-27 cells were captured using CLSM.

#### Intracellular ROS generation

Cal-27 and 4T1 cells (1 × 10^4^ cells per well) were seeded on multiple glass plates and incubated overnight to promote cell adhesion. The plates were then divided into seven groups: culture medium, laser, GA, HHPI@GA, HHHI@GA with laser (+ L), HHPI + L, and HHPI@GA + L, followed by a further 12-h culture period. The medium was replaced with fresh medium containing DCFH-DA (Ex/Em: 488/525 nm). After incubation for 30 min, cell imaging was performed by CLSM.

### Assessment of laser irradiation and HHPI cytotoxicity

The cytotoxic effects of blank HHPI NPs and laser irradiation on normal human liver cells (L-02 cells) and two cancer cell lines (Cal-27 and 4T1) were evaluated using a CCK-8 assay to avoid selective effects. Initially, a 96-well plate was inoculated with each cell type (3 × 10^3^ cells per well) and incubated overnight. Subsequently, 808 nm laser irradiation (1 W/cm^2^) was performed for 0–10 min per well, followed by replacement of the medium with the CCK-8 solution. The absorbance was measured at 450 nm.

Using the same method, cells were seeded in a 96-well plate and incubated overnight. The original medium was replaced with a medium containing various concentrations of HHPI. After further incubation for 48 h, the medium was replaced with the CCK-8 solution for analysis.

### In vitro anticancer evaluation

To avoid the selective effects of HHPI@GA, Cal-27 and 4T1 cancer cells were used to examine their anticancer effects. Cal-27 and 4T1 cells were seeded in various 96- and 6-well plates and incubated overnight to ensure cell attachment. The cells were then divided equally into seven groups: culture medium, laser, GA, HHPI@GA, HHHI@GA + L, HHPI + L, and HHPI@GA + L. Following treatment, the cells were incubated for an additional 12 h. The cytotoxicity of the various groups against cancer cells was assessed using live/dead staining and the CCK-8 and Annexin V-FITC/PI apoptosis assays. For live/dead staining, the cells were treated with Calcein-AM (Ex/Em: 495/515 nm) and PI (Ex/Em: 535/615 nm), and images were captured using CLSM. In the CCK-8 assay, after a 2-h treatment with the CCK-8 reagent, absorbance was measured at 450 nm using a microplate reader. Apoptosis was evaluated by co-staining the cells with Annexin V-FITC (Ex/Em: 488/525 nm) and PI and analyzing them via flow cytometry.

### Western blotting

Cal-27 cells were seeded in 6-well plates at a density of 1 × 10^5^ cells per well. Subsequently, consistent with the in vitro anticancer experiments, cells were divided into seven treatment groups. Next, the cells were collected and suspended in RIPA lysis buffer for protein extraction, and the total protein concentration was determined using a BCA protein assay kit. Proteins were separated using SDS-PAGE and transferred onto PVDF membranes. The membranes were blocked with 5% non-fat milk for 1 h, then incubated with primary antibodies overnight at 4 °C. Following three washes with TBST the next day, the membranes were incubated with secondary antibodies for 1 h at 25 °C. Immunoreactive bands were detected using an Enhanced Chemiluminescence Detection Kit (ProtoGlow ECL, National Diagnostics, USA) and imaged with a ChemiDoc™MP Imaging System (Bio-Rad, USA). The anti-HSP90 and GAPDH (control) antibodies were used for western blot analysis.

### Establishment of tumor models

All animal experiments were performed in accordance with the Ethical Committee of the Affiliated Hospital of Qingdao University and in line with the American Association for Laboratory Animal Science (AAALAS) guidelines.

The primary tumor was simulated using a subcutaneous tumor model. Cal-27 (2 × 10^6^/100 µL) and 4T1 cells (5 × 10^5^/100 µL) were subcutaneously injected into the right flank of 4-week-old male BALB/c nude mice and female BALB/c mice, respectively, thereby establishing the Cal-27 tumor model and the 4T1 tumor model.

Metastatic tumors were simulated in a lung metastasis mouse model. 4T1-luc cells (1 × 10^6^/200 µL) were injected intravenously into 6-week-old female BALB/c mice to establish a lung metastasis model. Three weeks post-inoculation, bioluminescence imaging based on 4T1-luc cells was performed to confirm the successful establishment of the lung metastasis model. After imaging, the mice were euthanized at random and their lung tissues were harvested for hematoxylin and eosin (H&E) staining.

### In vivo fluorescence and ^19^F MRI

#### Tumor-bearing mouse model

In the investigation of the in vivo and ex vivo biodistribution of HHPI@GA, upon the subcutaneous tumors attaining a volume of 50–100 mm^3^, free-ICG (Ex/Em: 785/820 nm), PP@ICG, and HHPI@GA were intravenously administered. In vivo fluorescence imaging was performed at designated intervals (3, 6, 12, 24, and 48 h) using an IVIS® Imaging System (PerkinElmer, USA). In addition, a BioSpec 94/30 USR 9.4 T (Bruker, Germany) small-animal MRI scanner was used to perform both ^1^H and ^19^F MRI at 24 h to confirm the in vivo biodistribution of HHPI@GA. Following euthanasia, major organs and tumors were collected for ex vivo imaging.

#### Lung metastasis mouse model

After successfully establishing the lung metastasis model, dual-modality imaging was performed to evaluate the in vivo biodistribution of HHPI@GA in mice with lung metastasis. The fluorescence imaging protocol was similar to that described above. The ^19^F MRI protocol was as follows: mice were administered intravenous injections of HHPI@GA. The mice were anesthetized and maintained with 2% isoflurane during scanning. ^1^H and ^19^F MR images were acquired at 6, 12, and 24 h post-injection. ^1^H MRI was performed using a RARE sequence: TR = 1000 ms, TE = 4.8 ms, matrix = 256 × 256, number of slices = 40, slice thickness = 1 mm, and FOV = 40 × 40 mm^2^. ^19^F MR images were collected in the same stereotaxic space as the ^1^H images, utilizing the following RARE sequence: TR = 1000 ms, TE = 4.62 ms, matrix = 32 × 32, slice thickness = 1 mm, FOV = 40 × 40 mm^2^, and acquisition time = 13 min 20 s.

### In vivo anticancer efficacy

Cal-27 and 4T1 tumor-bearing mice models were established for assessing the in vivo antitumor effects, as detailed in the ‘establishment of tumor models’ section. When the tumor volumes reached 50–100 mm^3^, mice were divided into seven groups (*n* = 5) and subjected to different treatments administered intravenously at 3 d intervals: saline; saline + L; GA; HHPI@GA; HHHI@GA + L; HHPI + L; and HHPI@GA + L. During irradiation, thermal images of the mice were captured using an IR thermal imager, and temperature measurements were taken at the tumor site every 30 s to confirm the PTT effect of HHPI@GA NPs. Body weight and tumor size were monitored twice daily. The tumor volumes were calculated as *V* = 1/2*a* × *b*^2^, where *V*, *a*, and *b* represent the volume, length, and width, respectively. On days 14 and 12, Cal-27 and 4T1 mice were euthanized for the collection and formaldehyde fixation (4%) of the major organs and tumor tissues. The collected subcutaneous tumors were measured, weighed, and photographed. The tumor inhibition rate was calculated using the following formula:

Inhibition rate (%) = [(mean tumor volume of control group − tumor volume of treatment group)/tumor volume of control group] × 100%.

Tumor samples underwent H&E staining and immunohistochemistry for Ki-67, TUNEL, and HSP90.

### Statistical analysis

Data are presented as mean ± standard deviation (SD). Statistical significance was determined using Student’s unpaired t-test or two-way analysis of variance (ANOVA), with significance thresholds set at *P < 0.05, **P < 0.01, ***P < 0.001, and ****P < 0.0001.

**.**
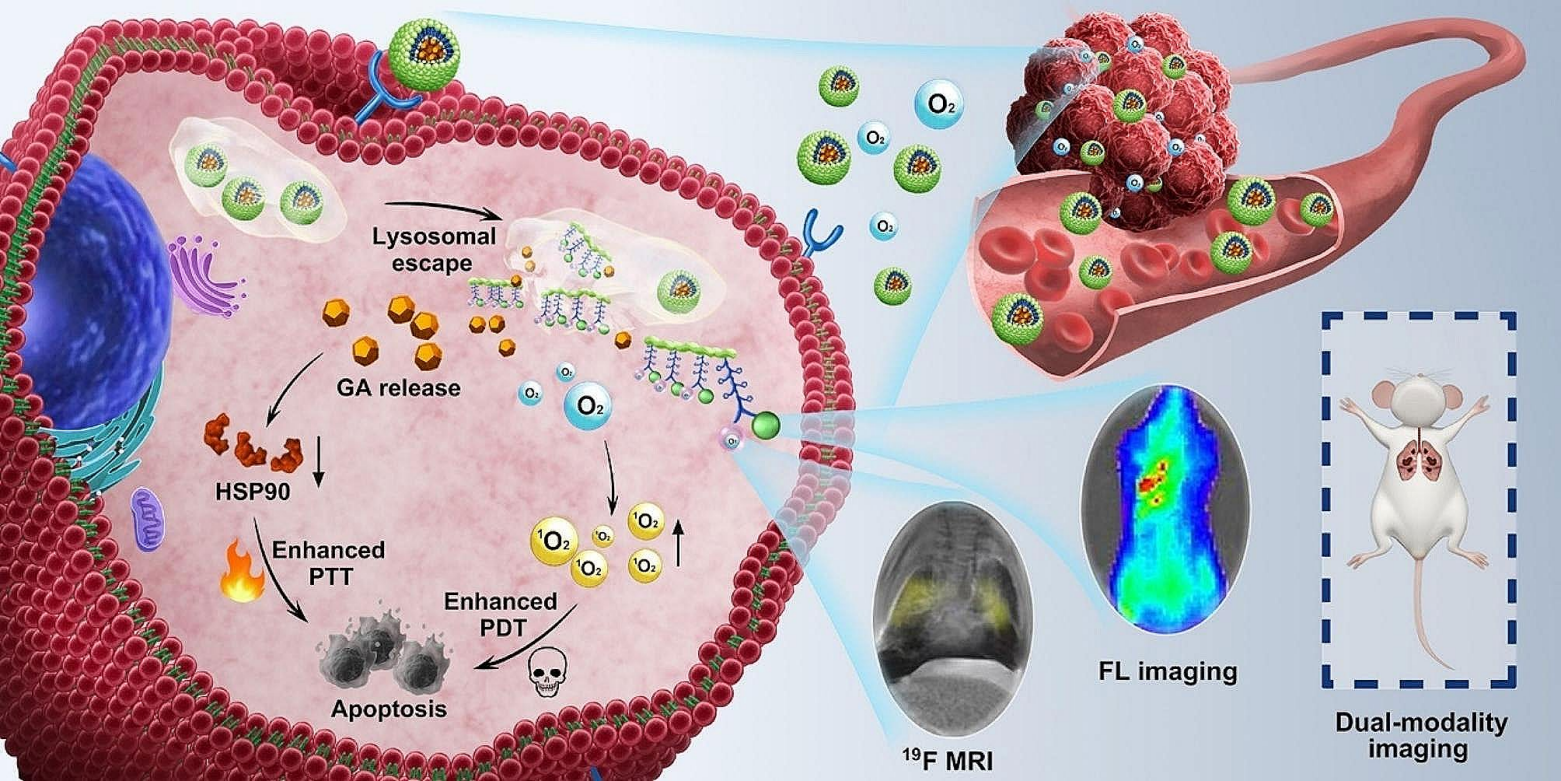
In this work, a pH-responsive dual-modality imaging nanoplatform, HHPI@GA, was designed to overcome key challenges in conventional phototherapy for cancer treatment, offering a promising strategy for efficient enhanced phototherapy and metastasis diagnosis, and displaying broad clinical application prospects.

### Electronic supplementary material

Below is the link to the electronic supplementary material.

Supplementary Material 1

## Data Availability

No datasets were generated or analysed during the current study.

## Abbreviations

PTT: photothermal therapy
PDT: photodynamic therapy
NP: nanoplatform
MRI: magnetic resonance imaging
H_6_: hexahistidine
HHPI: HA-H_6_-PFC-ICG
HHHI: HA-H_6_-He-ICG
GA: gambogic acid
HA: hyaluronic acid
ROS: reactive oxygen species
HSP: heat shock protein
TME: tumor microenvironment
ICG: indocyanine green
NIR: near-infrared
FDA: Food and Drug Administration
DI: deionized
EPR: enhanced permeability and retention
SNR: signal-to-noise ratio
CLSM: confocal laser scanning microscopy
TIR: tumor inhibition rate
HA-SH: hyaluronic acid-thiol
PP: mPEG-PCL
PFC: 1 H,1 H-Undecafluorohexylamine
He: 1-Hexadecylamine
Mal-COOH: 3-MaleimDaidopropionic acid
HATU: O-benzotriazole-N, N, N’, N’-tetramethyluronium hexafluorophosphate
DIPEA: N, N-diisopropylethylamine
NHS: N-hydroxysuccinimide
EDC·HCl: N-(3-dimethylaminopropyl)-N′-ethylcarbondiimine hydrochloride
DMF: anhydrous N, N-dimethylformamide
DMEM: Dulbecco’s modified Eagle’s medium
FBS: fetal bovine serum
DCFH-DA: 2’,7’-dichlorodihydrofluorescein diacetate
CCK-8: cell counting kit-8
GAPDH: glyceraldehyde-3-phosphate dehydrogenase
GPC: gel permeation chromatography
FTIR: fourier-transform infrared spectroscopy
PDI: polydispersity index
DLS: dynamic light scattering
TEM: transmission electron microscopy
DLC: drug-loading capacity
EE: encapsulation efficiency
CMC: critical micelle concentration
RBC: red blood cell
NMR: nuclear magnetic resonance
H&E: hematoxylin and eosin
