# Supplementary material for: A pH-responsive nanoplatform with dual-modality imaging for enhanced cancer phototherapy and diagnosis of lung metastasis
Source: J Nanobiotechnology. 2024 Apr 15;22:180. doi: 10.1186/s12951-024-02431-6 (PMC11017640; doi:10.1186/s12951-024-02431-6)
Supplement: Supplementary file 1 — Supplementary Material 1 [file 12951_2024_2431_MOESM1_ESM.docx]

**Supporting information**

A pH-responsive nanoplatform with dual-modality imaging for enhanced cancer phototherapy and diagnosis of lung metastasis

Mujie Yuan^1†^, Zeyu Han^1†^, Yan Li^2†^, Xin Zhan^1^, Yong Sun^3^, Bin He^4^, Yan Liang^3^*, Kui Luo^5^ and Fan Li^1,3^*

^1^Department of Oral Implantology, The Affiliated Hospital of Qingdao University, Qingdao 266000, China

^2^Precision Research Center for Refractory Diseases, Shanghai General Hospital, Shanghai Jiao Tong University School of Medicine, Shanghai 200080, China

^3^Department of Pharmaceutics, Qingdao University School of Pharmacy, Qingdao 266021, China

^4^National Engineering Research Center for Biomaterials, Sichuan University, Chengdu 610064, China

^5^ Huaxi MR Research Center (HMRRC), Department of Radiology, West China Hospital, Sichuan University, Chengdu 610041, China

^†^These authors contributed equally in this work.
* To whom correspondence should be addressed, E-mail: [liangyan072@foxmail.com](mailto:liangyan072@foxmail.com) (Y. Liang); [lifan911017@qdu.edu.cn](mailto:lifan911017@qdu.edu.cn) (F. Li)

**TABLE OF CONTENTS**

**SUPPLEMENTAL FIGURES**

**Figure S1**…….………….…………….……….………..……….…...…….…….………..……….3

**Figure S2**…….…………….……….……….………………….……...………….………..…….4

**Figure S3**…….…………….……….……….…….….….……………………….………………5

**Figure S4**…….………….…….……….………..…….….……….…………..….………………6

**Figure S5**…….………….…….……….………….……….…………………….………………7

**Figure S6**…….………………….………..….…….……...….………………….………………8

**Figure S7**…….…………………….….…….……….……….……….…….…...………………9

**Figure S8**…….……………………………….………….……….…….….……….….………….10

**Figure S9**…….……………………………….………….………..……….…………..……….....11

**Figure S10**……..…………………………….…………..……….……….……….…..………….12

**Figure S11**……..…………………………….…………..……….……….……….…..……….....13

**Figure S12**……..…………………………….…………..……….……….……….…..……...…..14

**Figure S13**……..…………………………….…………..……….……….………...………...…..15

**Figure S14**……..……………………….…….…………..……….……….……….…..…….…...16

**Figure S15**……..…………………………….…………..……….……….……….…..……...….17

**Figure S16**……..…………………………….…………..……….……….……….…..……...…..18

**Figure S17**……..…………………………….…………..……….……….……….…..……...…..19

**Figure S18**……..…………………………….…………..……….……….……….…..……...…..20


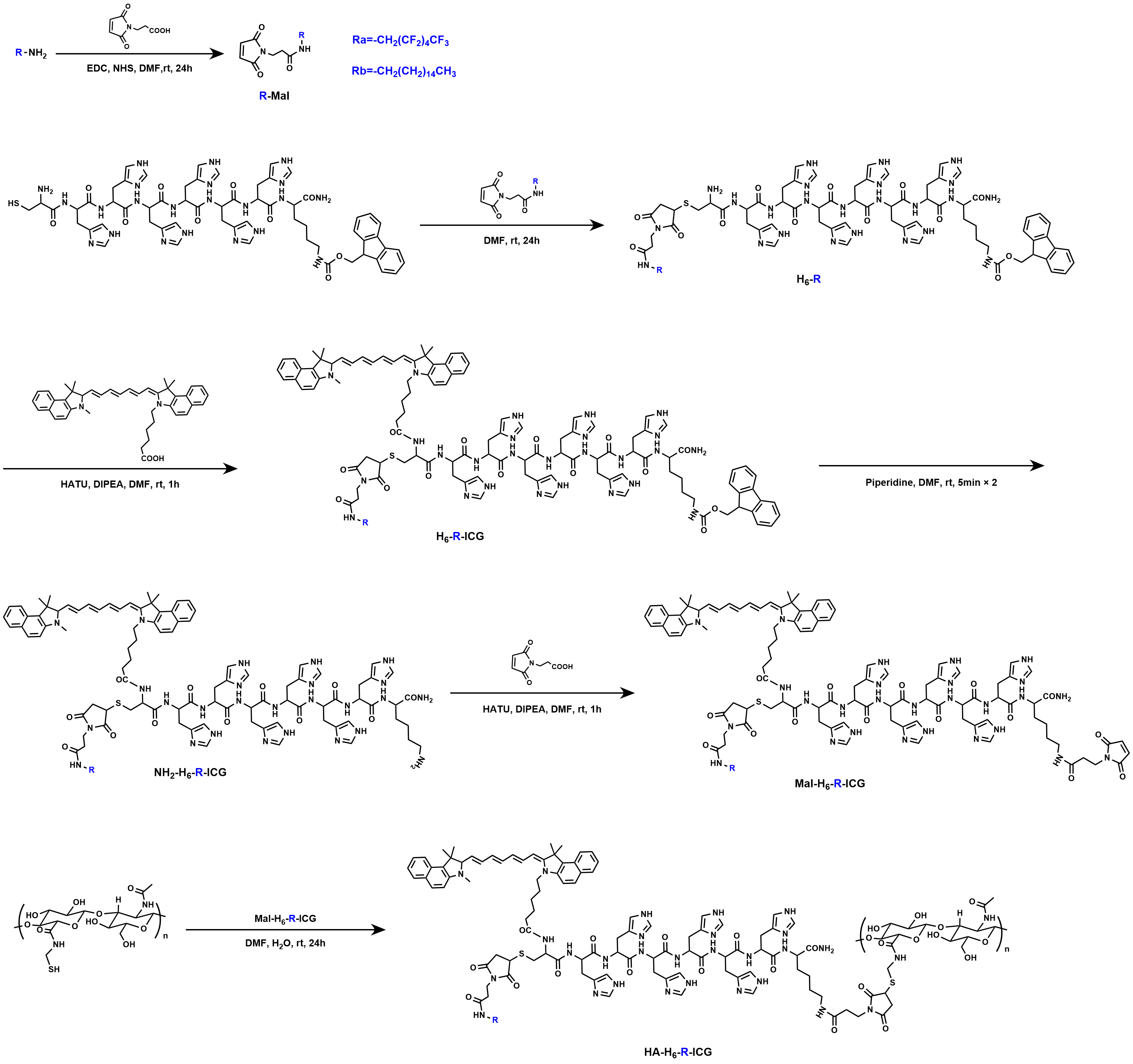


**Fig. S1.** The synthesis scheme of HHPI (Ra) and HHHI (Rb).


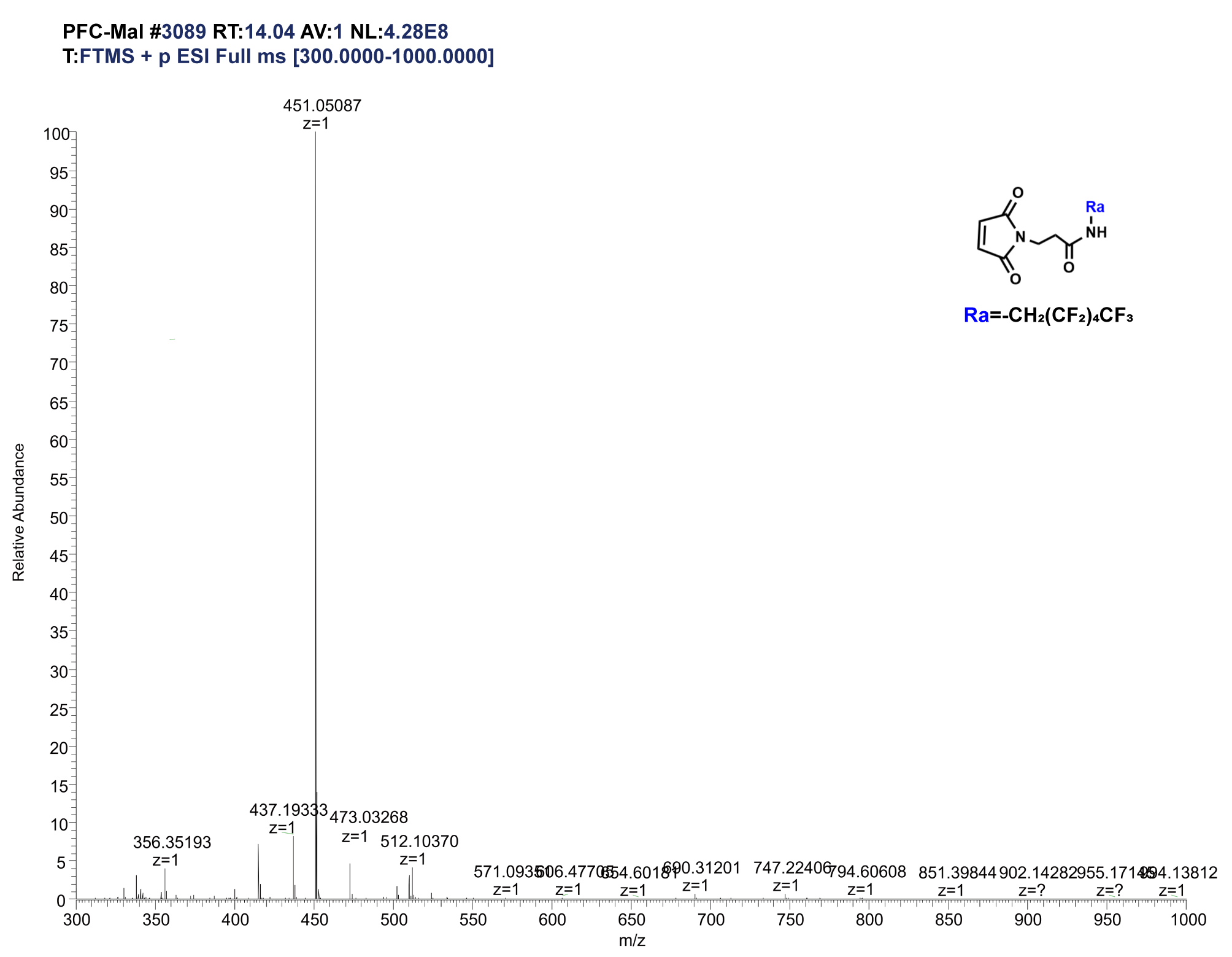


**Fig. S2.** Chemical structure and ESI-TOF mass spectrum of PFC-Mal, Exact mass: 450.04375 (calculated), m/z [M+H]^+^: 451.05087 (observed).


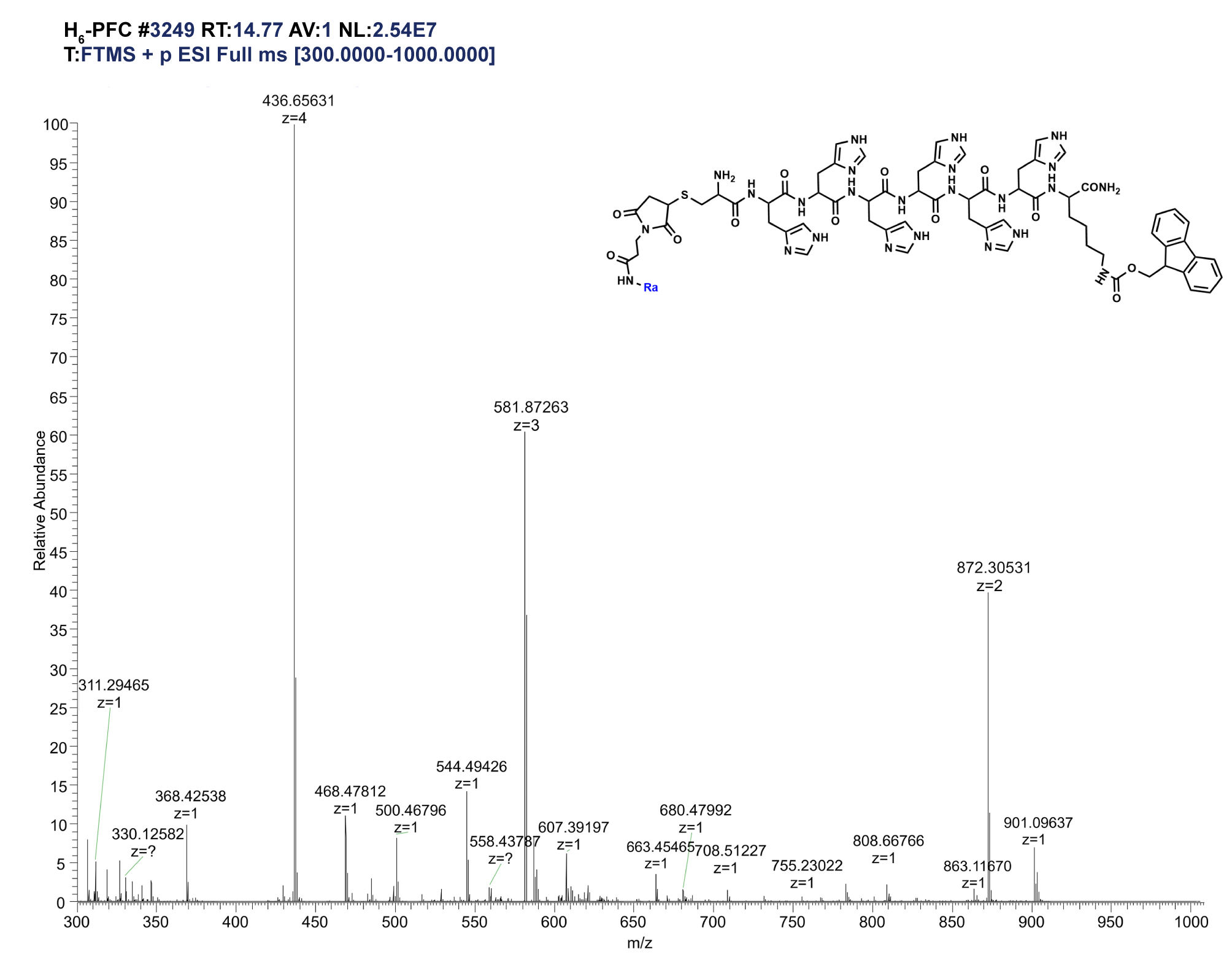


**Fig. S3.** Chemical structure and ESI-TOF mass spectrum of H_6_-PFC, Exact mass: 1742.596 (calculated), m/z [M+2H]^2+^: 872.30531; [M+3H]^3+^: 581.87263; [M+4H]^4+^: 436.65631 (observed).


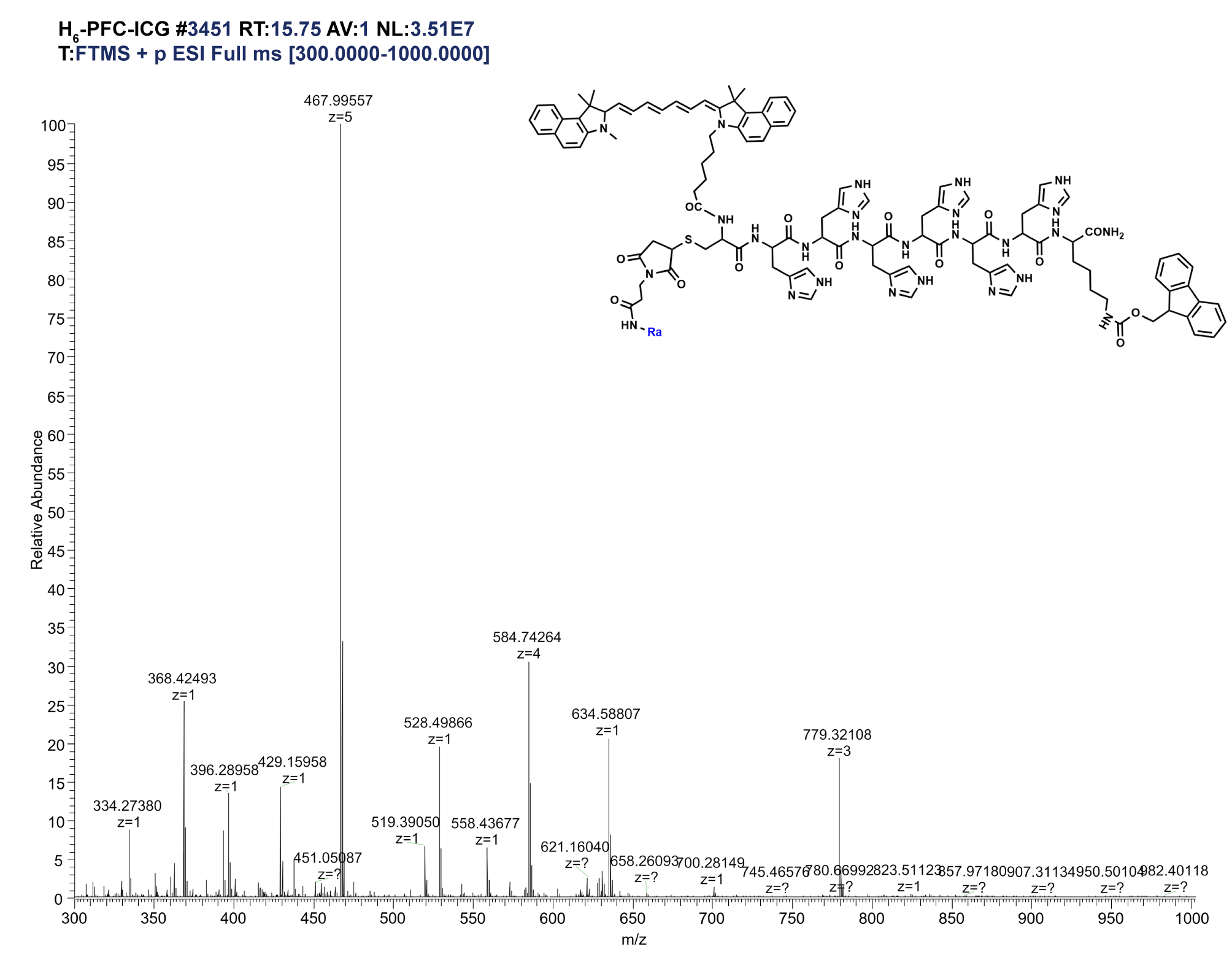


**Fig. S4.** Chemical structure and ESI-TOF mass spectrum of the H_6_-PFC-ICG, Exact mass: 2334.94136 (calculated), m/z [M+3H]^3+^: 779.32108; [M+4H]^4+^: 584.74264; [M+5H]^5+^: 467.99557 (observed).


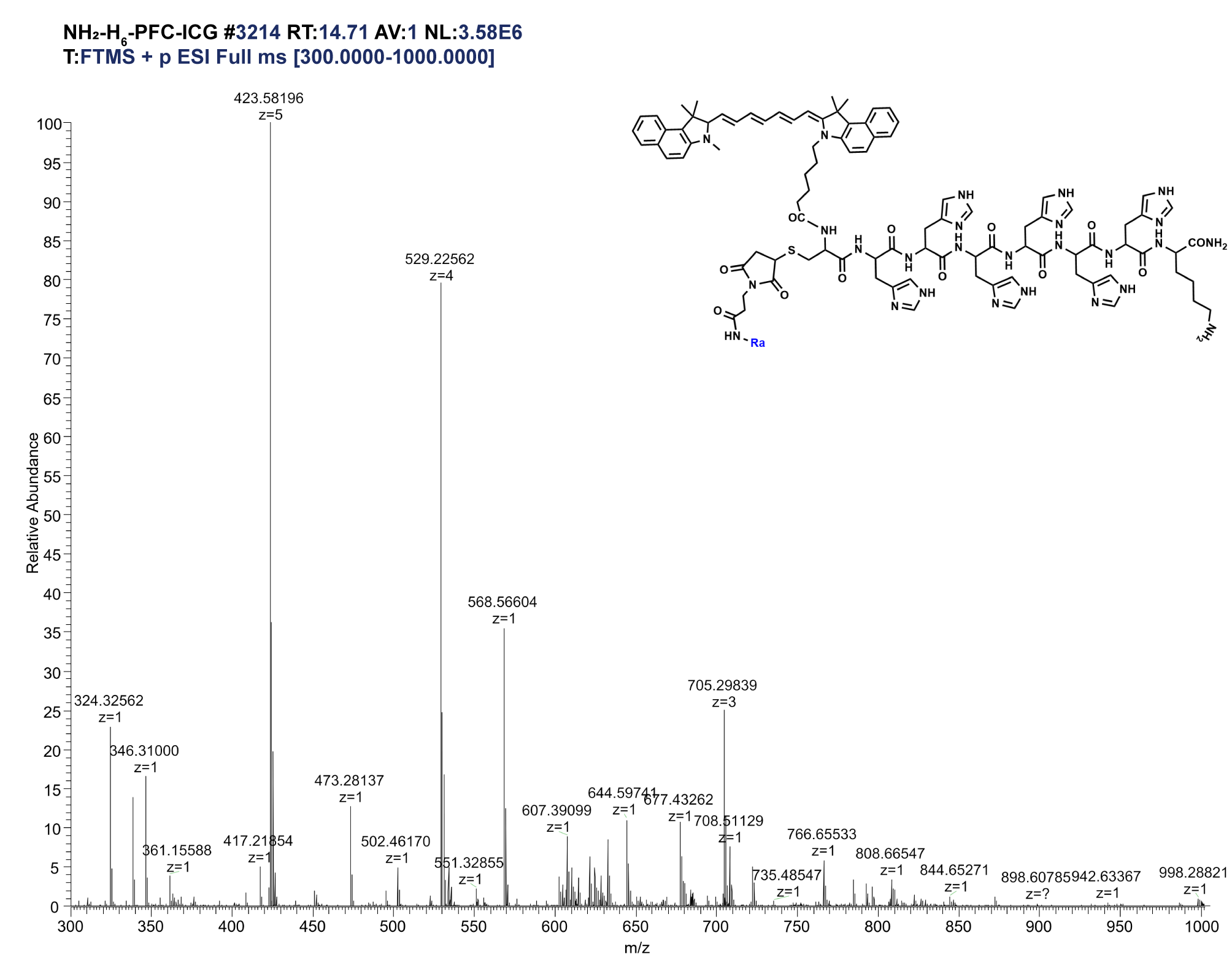


**Fig. S5.** Chemical structure and ESI-TOF mass spectrum of the NH_2_-H_6_-PFC-ICG, Exact mass: 2112.87328 (calculated), m/z [M+3H]^3+^: 705.29839; [M+4H]^4+^: 529.22562; [M+5H]^5+^: 423.58196 (observed).


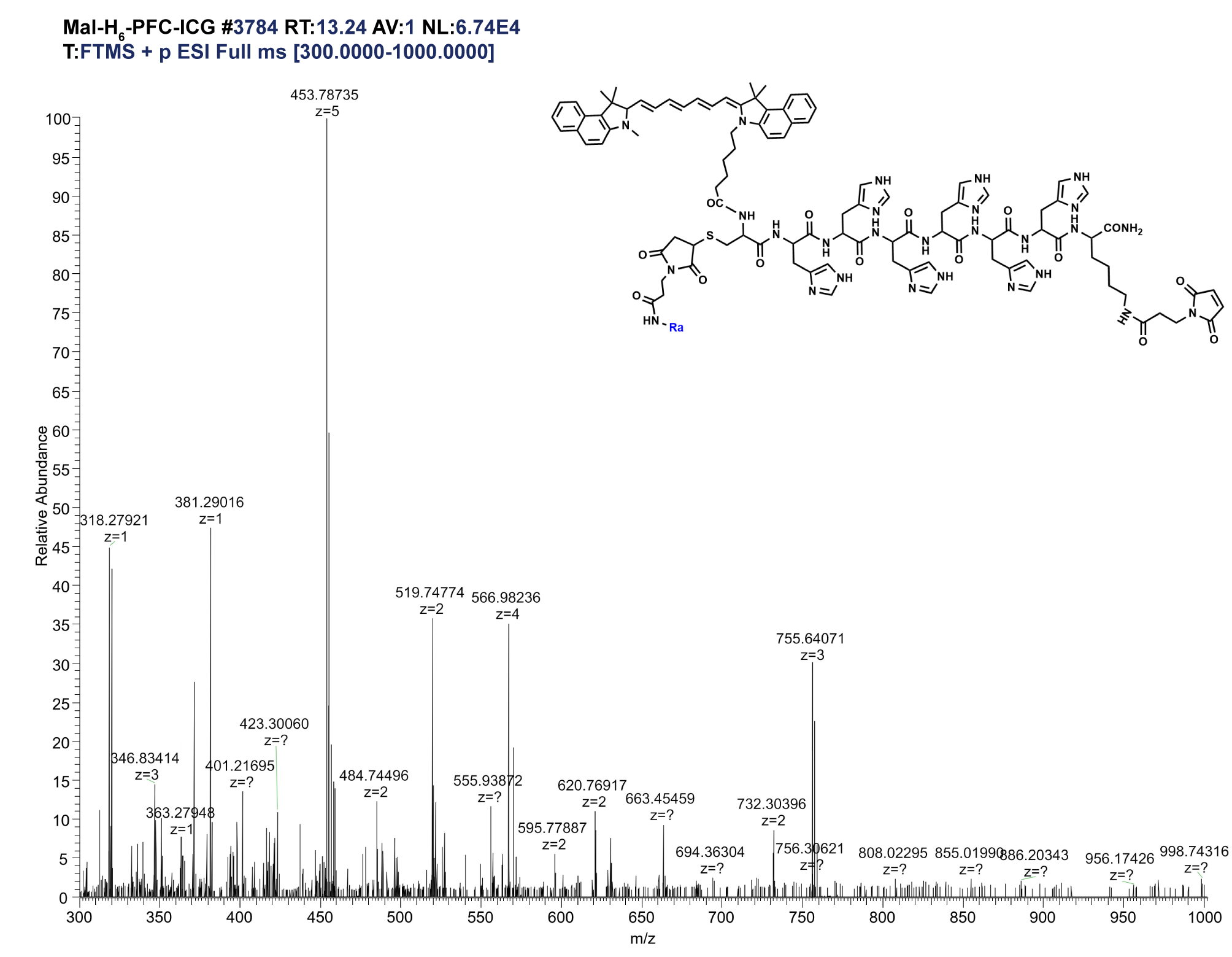


**Fig. S6.** Chemical structure and ESI-TOF mass spectrum of the Mal-H_6_-PFC-ICG, Exact mass: 2263.90023 (calculated), m/z [M+3H]^3+^: 755.64071; [M+4H]^4+^: 566.98236; [M+5H]^5+^: 453.78735 (observed).


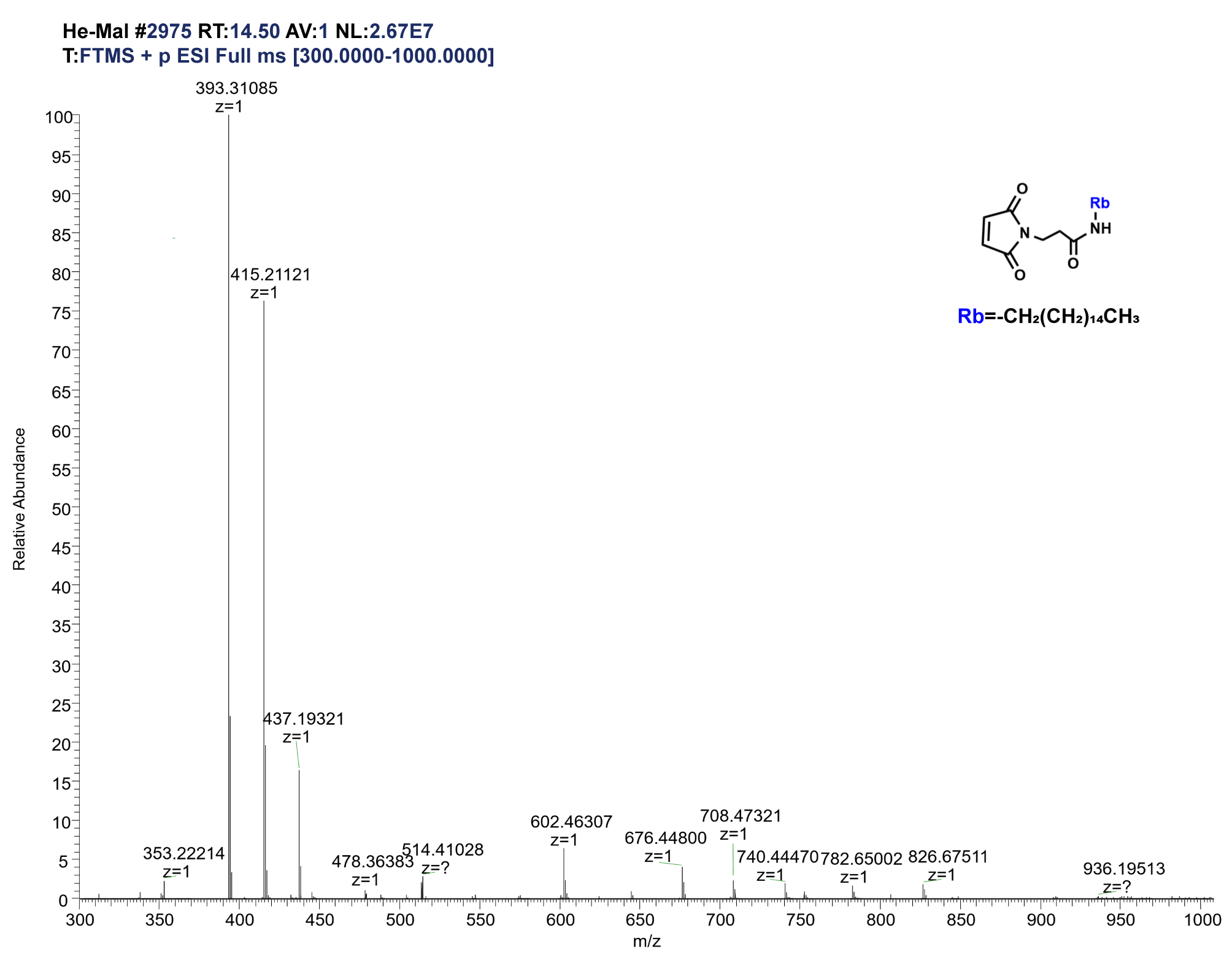


**Fig. S7.** Chemical structure and ESI-TOF mass spectrum of the He-Mal, Exact mass: 392.30389 (calculated), [M+H]^+^: 393.31085, [M+Na]^+^: 415.21121(observed).


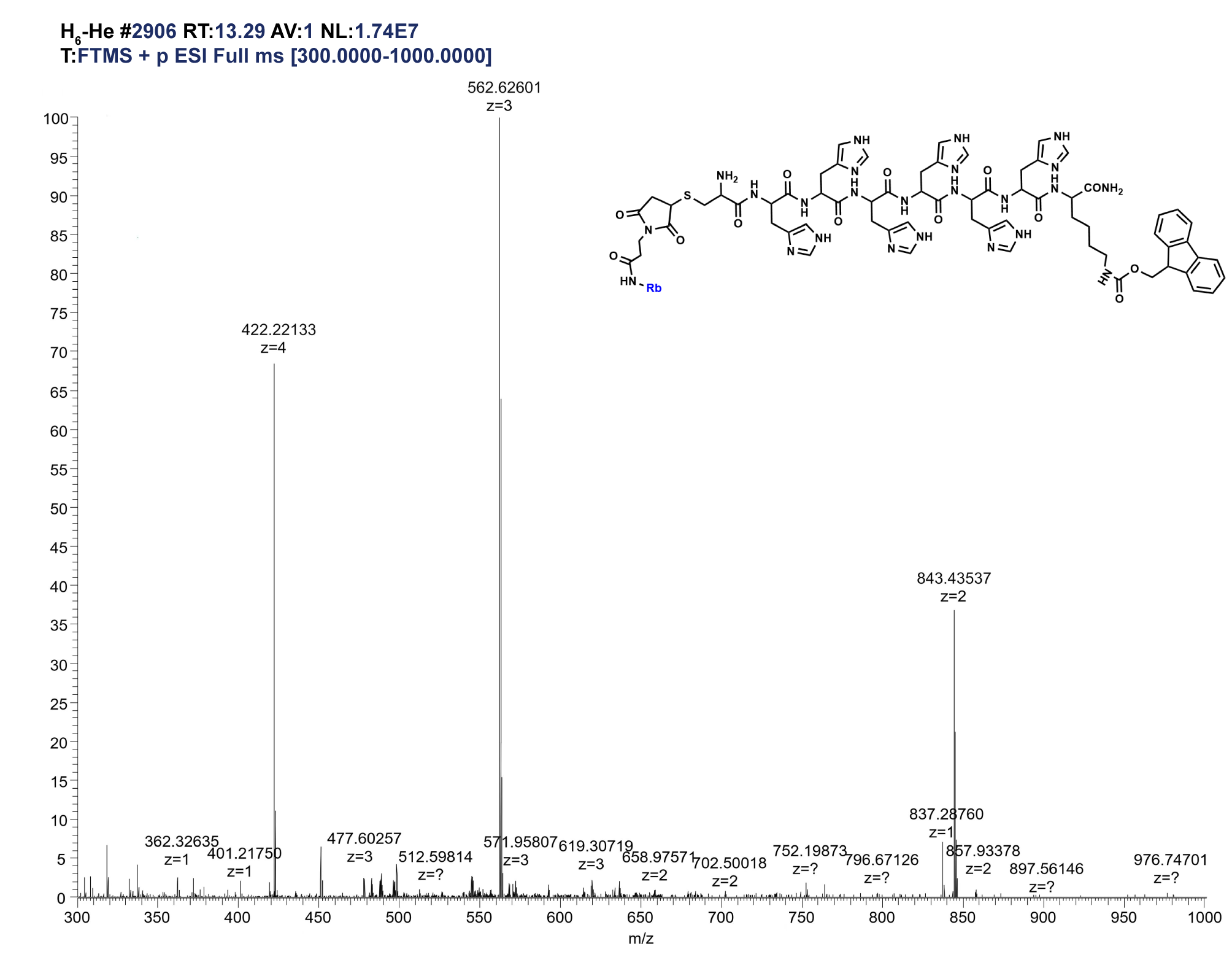


**Fig. S8.** Chemical structure and ESI-TOF mass spectrum of the H_6_-He, Exact mass: 1684.85614(calculated), m/z [M+2H]^2+^: 843.43537; [M+3H]^3+^: 562.62601; [M+4H]^4+^: 422.22133 (observed).


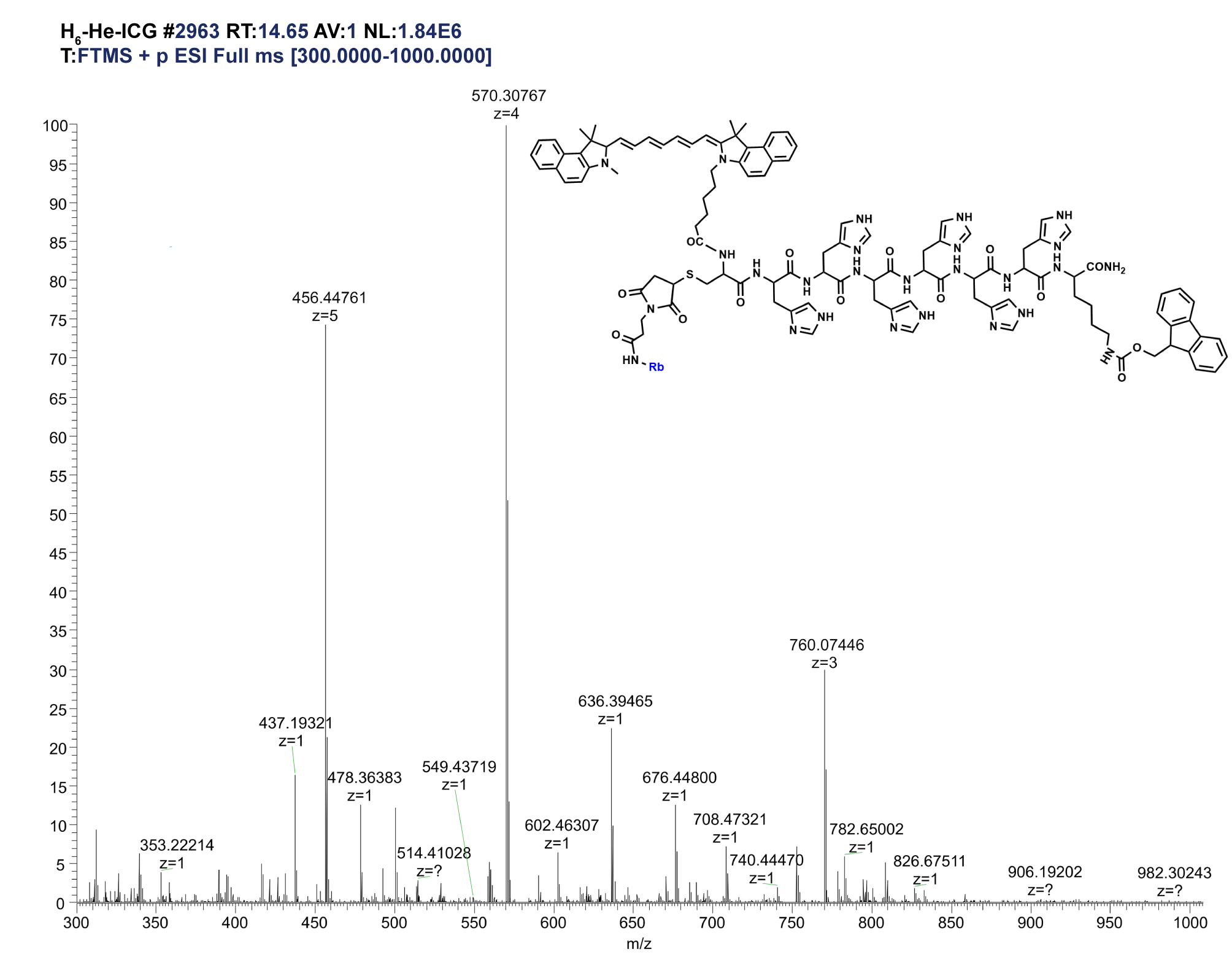


**Fig. S9.** Chemical structure and ESI-TOF mass spectrum of the H_6_-He-ICG, Exact mass: 2277.2015 (calculated), m/z [M+3H]^3+^: 760.07446; [M+4H]^4+^: 570.30767; [M+5H]^5+^: 456.44761 (observed).

**
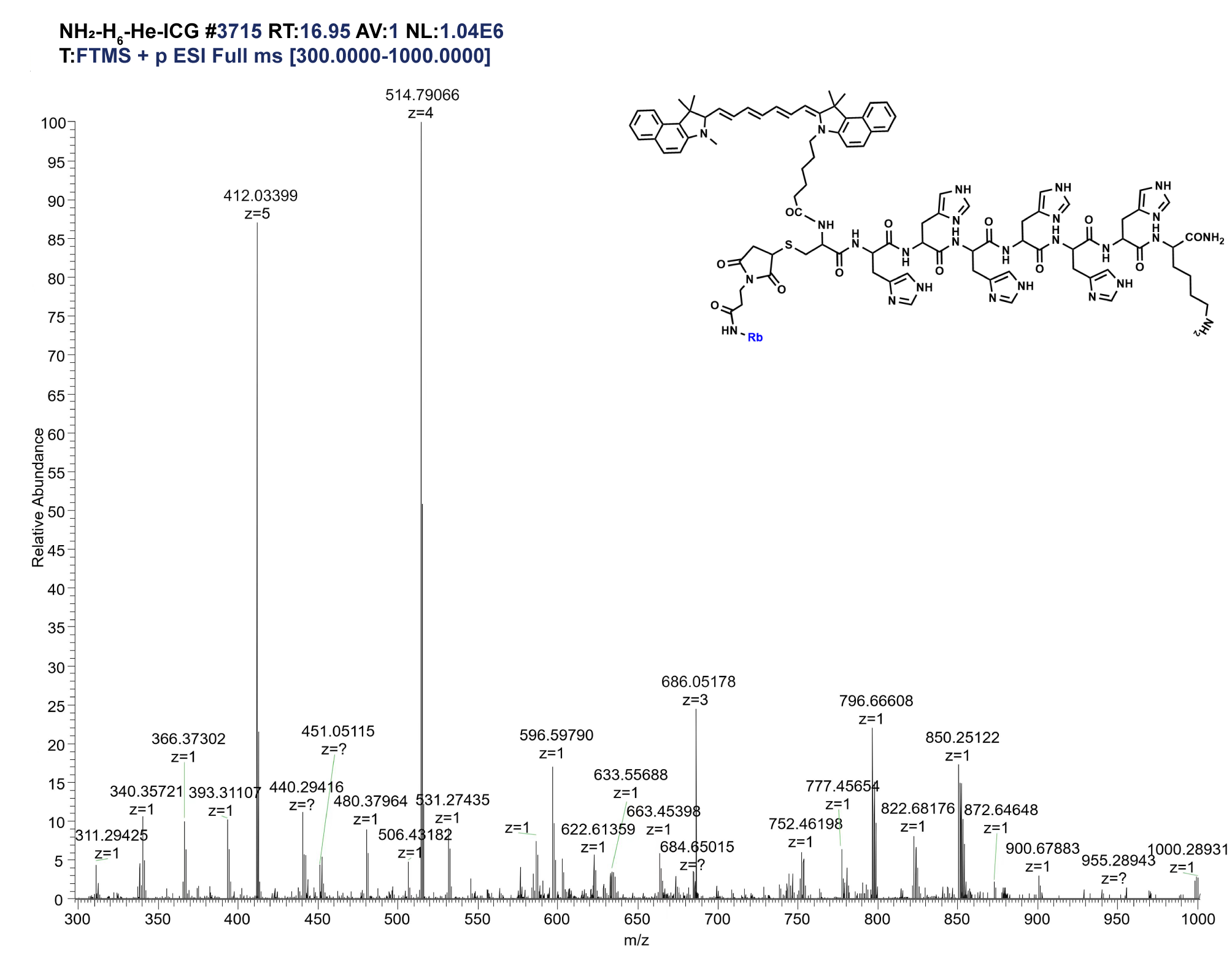
**

**Fig. S10.** Chemical structure and ESI-TOF mass spectrum of the NH_2_-H_6_-He-ICG, Exact mass: 2055.13343 (calculated), m/z [M+3H]^3+^: 686.05178; [M+4H]^4+^: 514.79066; [M+5H]^5+^: 412.03399 (observed).


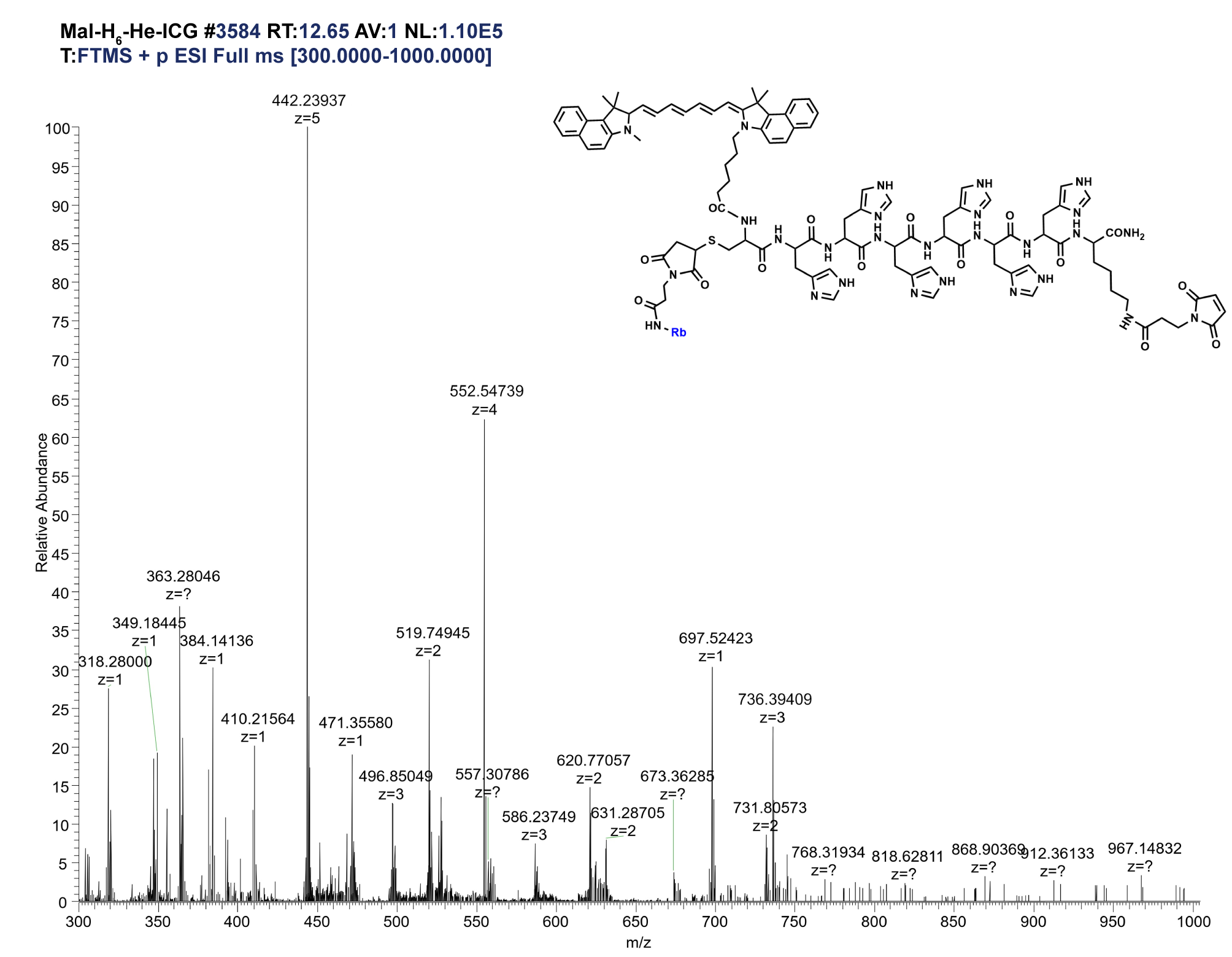


**Fig. S11.** Chemical structure and ESI-TOF mass spectrum of the Mal-H_6_-He-ICG, Exact mass: 2206.16037 (calculated), m/z [M+3H]^3+^: 736.39409; [M+4H]^4+^: 552.54739; [M+5H]^5+^: 442.23937 (observed).


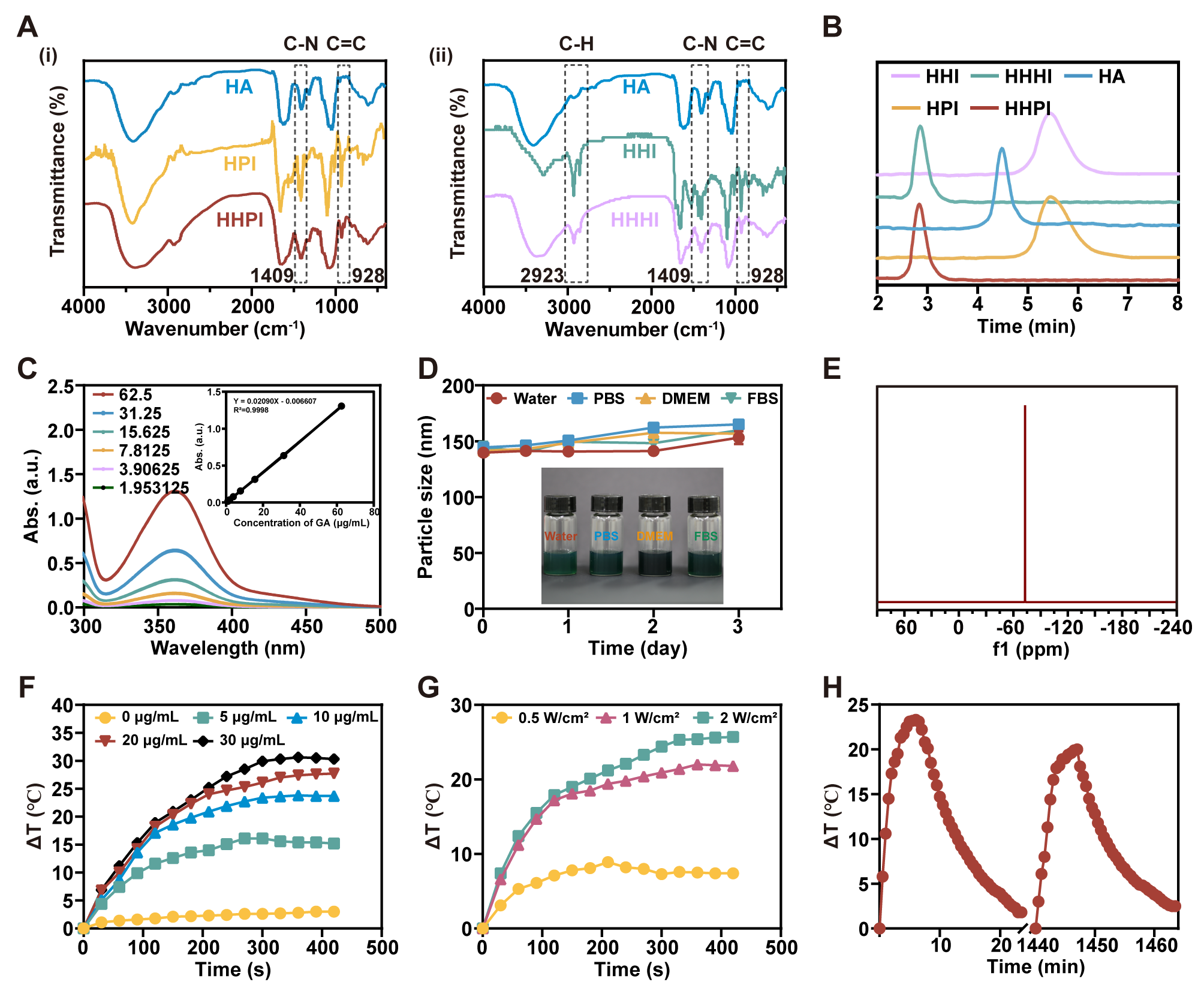


**Fig. S12. A.** Infrared spectroscopy of HA, HPI, and HHPI **(i)**, and HA, HHI, and HHHI **(ii)**. **B.** GPC spectrum of HHI, HHHI, HA, HPI, and HHPI. **C.** Standard line of GA measured by UV-vis spectrophotometer. **D.** Size changes and photos of HHPI@GA in different solutions over 3 days. **E.** ^19^F NMR spectrum (DMSO-d_6_) of HHPI@GA NPs. **F.** Temperature curves of HHPI@GA at different concentrations under laser irradiation (1 W/cm^2^). **G.** Temperature curves of HHPI@GA under different power density irradiations. **H.** The photothermal capability of HHPI@GA NPs over two laser on/off cycles in a 24 h interval of incubation.


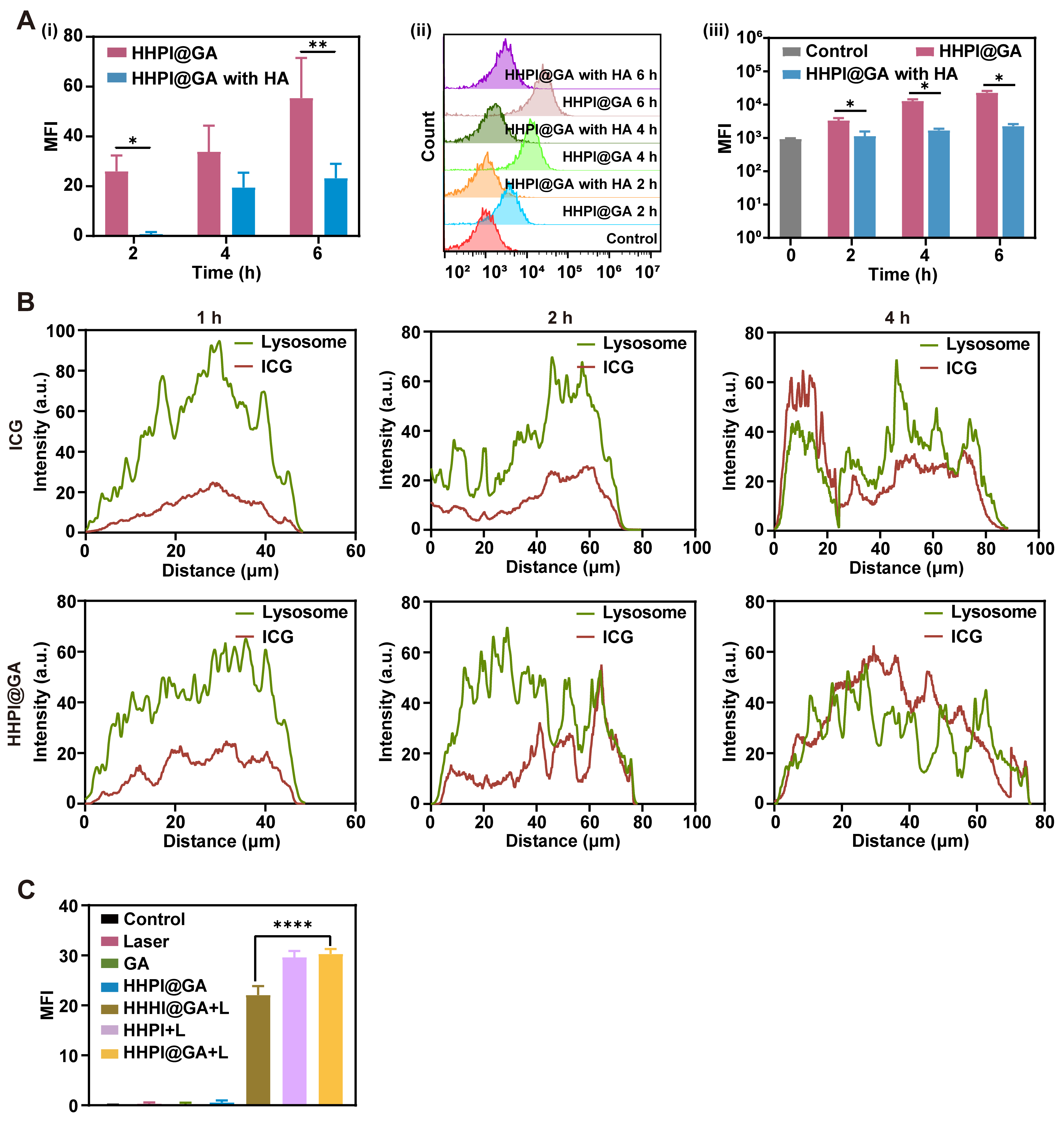


**Fig. S13. A.** Quantitative analysis of CLSM images (i), flow cytometry (ii) and its corresponding mean fluorescence intensities (iii) of cell uptake in Cal-27 cells. **B.** Colocalization analysis of lysosome escape of free ICG and HHPI@GA NPs. **C.** Quantitative analysis of CLSM images of ROS detection in Cal-27 cells. Data are shown as mean ± SD (n = 3) (**P* < 0.05, ***P* < 0.01 and *****P* < 0.0001).


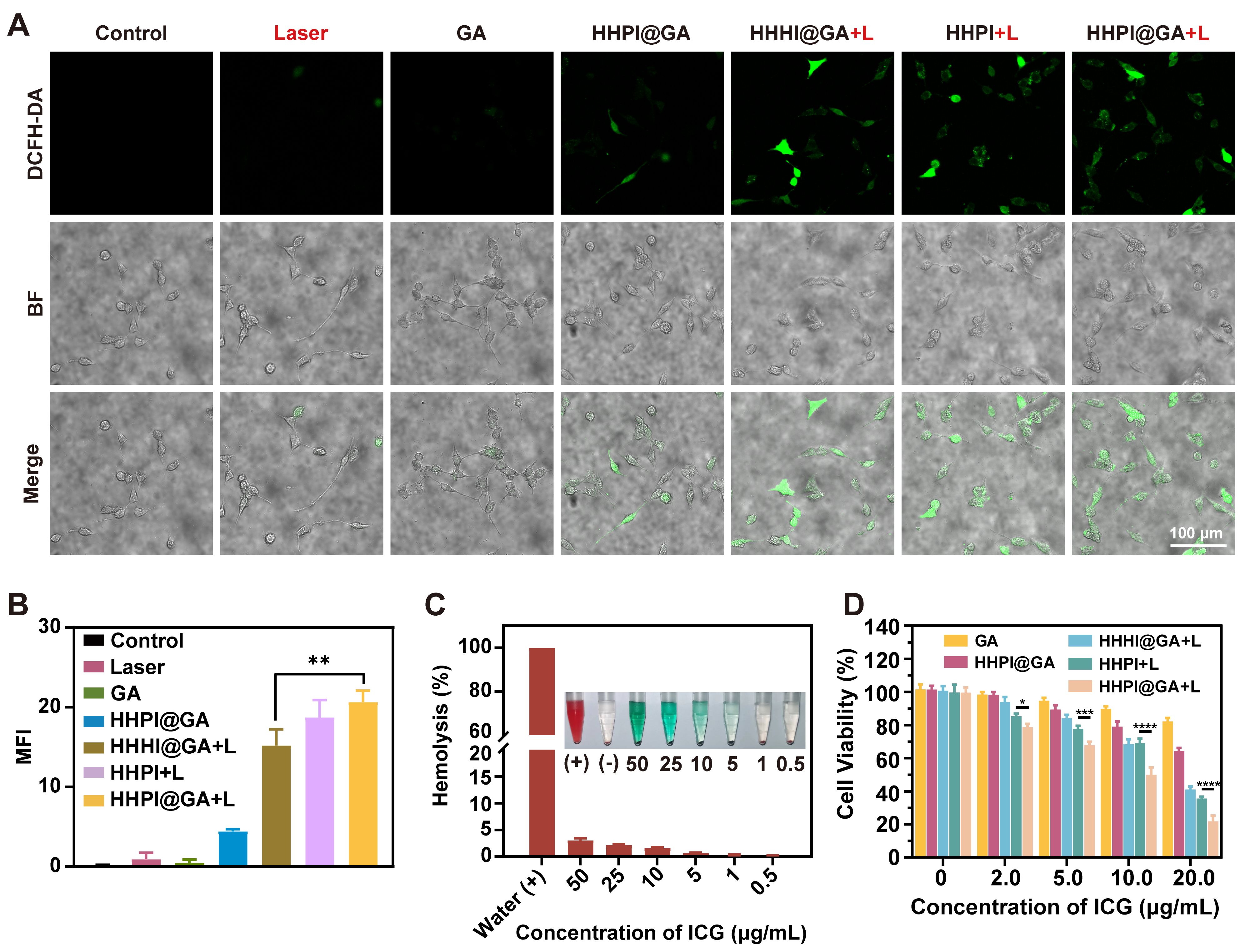


**Fig. S14. A, B.** ROS generation *in vitro* CLSM images after different treatments of 4T1 cells **(A)** and quantitative analysis**(B)**. Scale bar: 100 μm. **C**. Hemolysis rate and photos of HHPI at different concentrations. **D.** Cell viability of 4T1 cells after various treatments. Data are shown as mean ± SD (n = 3) (**P* < 0.05, ***P* < 0.01, ****P* < 0.001, and *****P* < 0.0001).


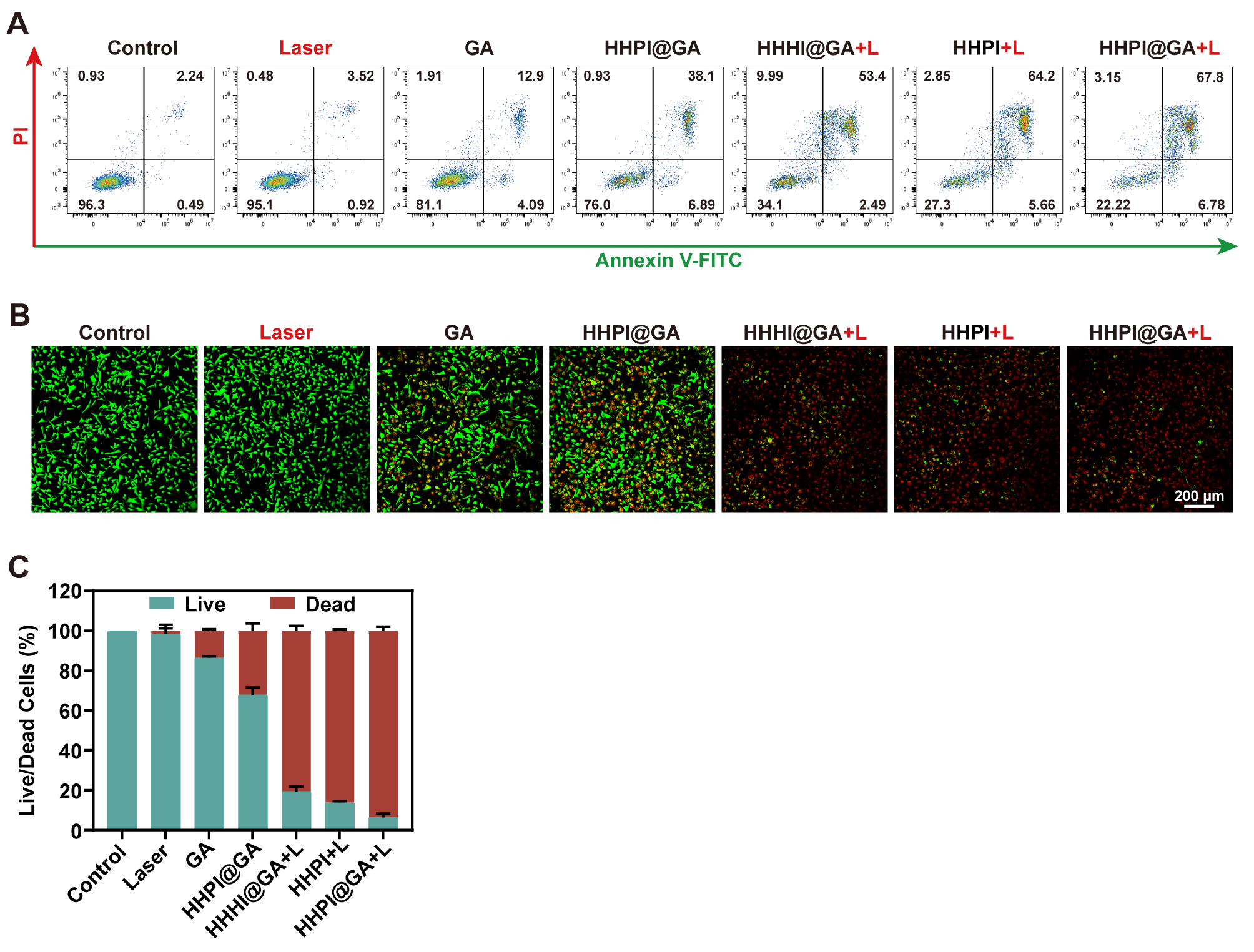


**Fig. S15. A.** Apoptosis analysis of 4T1 cells after various treatments. **B, C**. CLSM images **(B)** and semi-quantitative analysis **(C)** of live/dead cell assays of 4T1 cells. Scale bar: 200 μm.


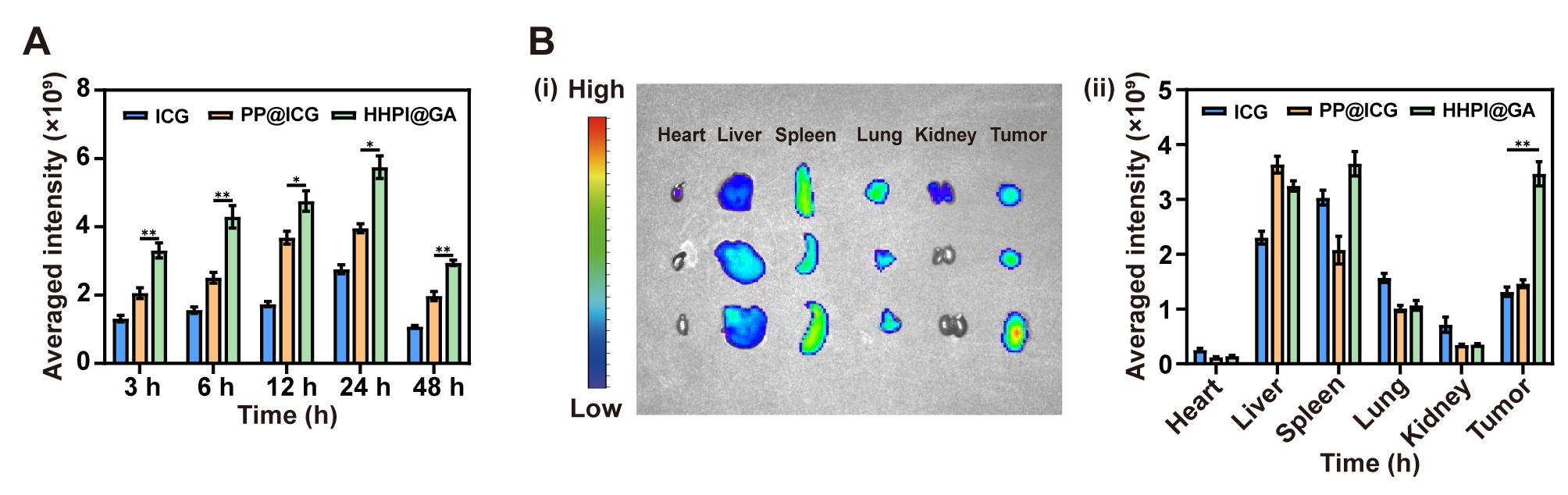


**Fig. S16. A.** Real-time average radiant efficiency of tumor in subcutaneous tumor mice. **B.** *Ex vivo* fluorescence images **(i)** and average radiant efficiency **(ii)** of major organs and tumor at 48 h after intravenous injection of different solutions. Data are shown as mean ± SD (n = 3) (**P* < 0.05 and ***P* < 0.01).


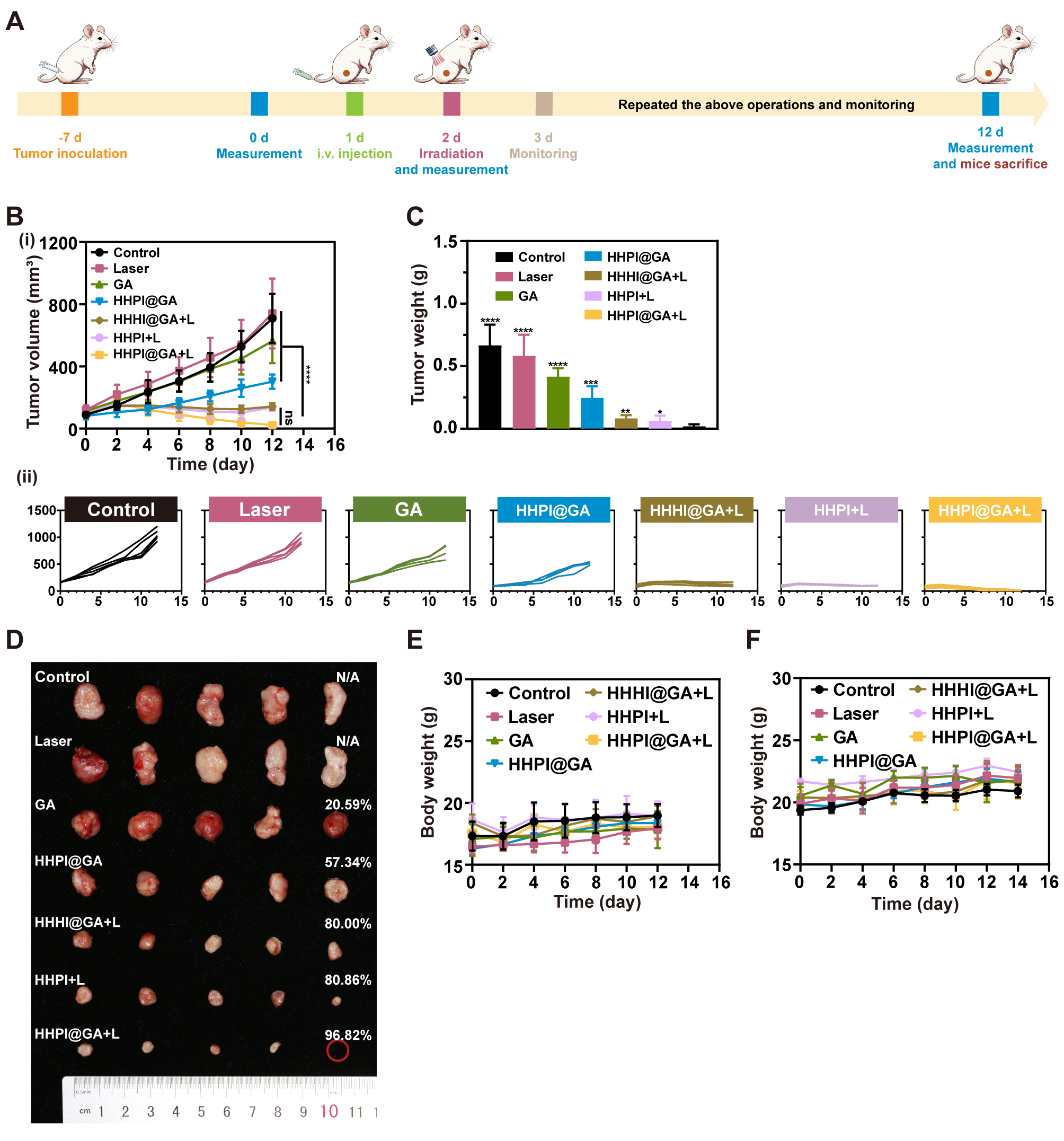


**Fig. S17. A.** Schematic of treatments in 4T1 tumor-bearing mice. **B.** Tumor volume **(i)** and tumor growth curves **(ii)** of 4T1 tumor-bearing mice. **C.** tumor weights of different treatment groups in 4T1 tumor-bearing mice. **D.** Photographs and TIR values of tumors after 12-day treatments. **E, F.** Body weight curves of different treatment groups in 4T1 **(E)** and Cal-27 **(F)** tumor-bearing mice. Data are shown as mean ± SD (n = 5) (**P* < 0.05, ***P* < 0.01, ****P* < 0.001, and *****P* < 0.0001).


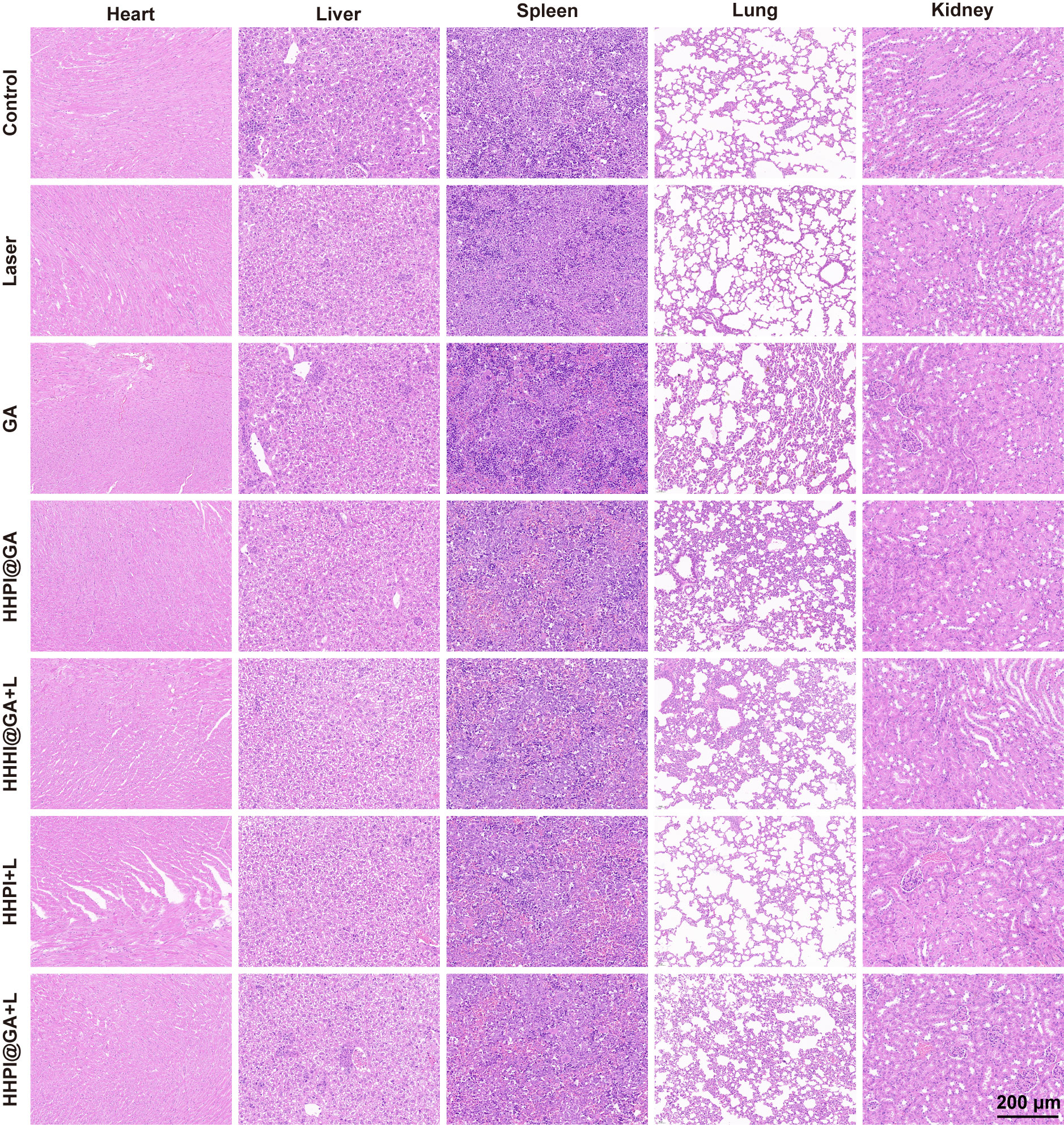


**Fig. S18.** The histological analyses of major organs of subcutaneous tumor mice by H&E staining. Scale bar: 200 μm.
